# Apple leaf disease image recognition based on a modified rime optimization algorithm and ConvNeXt network

**DOI:** 10.3389/fpls.2025.1626335

**Published:** 2025-09-10

**Authors:** Jing Qian, Linjing Wei

**Affiliations:** College of Information Science and Technology, Gansu Agricultural University, Lanzhou, China

**Keywords:** apple leaf disease recognition, ConvNeXt architecture, modified rime optimization algorithm, attention mechanism, data augmentation, precision agriculture

## Abstract

Early and accurate diagnosis of apple leaf disease is a prerequisite for maintaining crop health and for enhancing agricultural productivity. Conventional methods, which largely relied on human inspection or naive machine learning algorithms, were not capable of handling the complexity of patterns, the class imbalance, and the real-world challenges such as conflated symptoms or poor lighting. The present study develops a completely new model design by integrating a ConvNeXt model along with a modified rime optimization algorithm (MRIME) used for hyperparameter tuning as well as complementing through the Convolutional Block Attention Module (CBAM) to ensure better feature extraction. CBAM extends the power of the model in focusing on critical discriminative regions, while MRIME gives optimal values for relevant hyperparameters for generalization while avoiding overfitting. Evaluated by the Apple Leaf Disease Symptoms Dataset, the proposed approach attained an accuracy of 92.7%, precision of 92.5%, recall of 92.6%, F1-score of 92.5%, and mAP of 92.3%, surpassing most baselines including ResNet50 and EfficientNet-B0. Compared to the aforementioned baselines, ablation experiments demonstrated that CBAM led to about 1.5% enhancement in accuracy, while MRIME could boost performance by another 1.2% via hyperparameter tuning. These results confirm the complementary benefit of attention mechanisms and metaheuristic optimization in producing state-of-the-art results.

## 1 Introduction

Apple production is one of the pillars of global agriculture and makes a significant contribution to food security, rural lives, and economic development (Zou et al., 2025). Apple fruits are highly vulnerable to diseases caused by infection by pathogens such as fungi, bacteria, and viruses. Some of the commonly known diseases include apple scab, powdery mildew, rust, and black rot, which all have special symptoms on the leaves, fruits, or stems. These diseases, apart from reducing crop production, also reduce the quality of fruits and cause significant economic loss to farmers. Early detection and accurate diagnosis of the diseases are of the highest priority for a number of reasons, namely:

1. Prevention of spread of disease: It can prevent the spread of disease to other nearby plants, which helps in preventing crop loss.
2. Pesticide reduction: Accurate disease identification enables site-specific pesticide application, reducing the environmental influence and economic costs.
3. Increased crop yield: Early treatment guarantees healthier crops, resulting in increased yields and improved-quality production.

**Figure 1:**
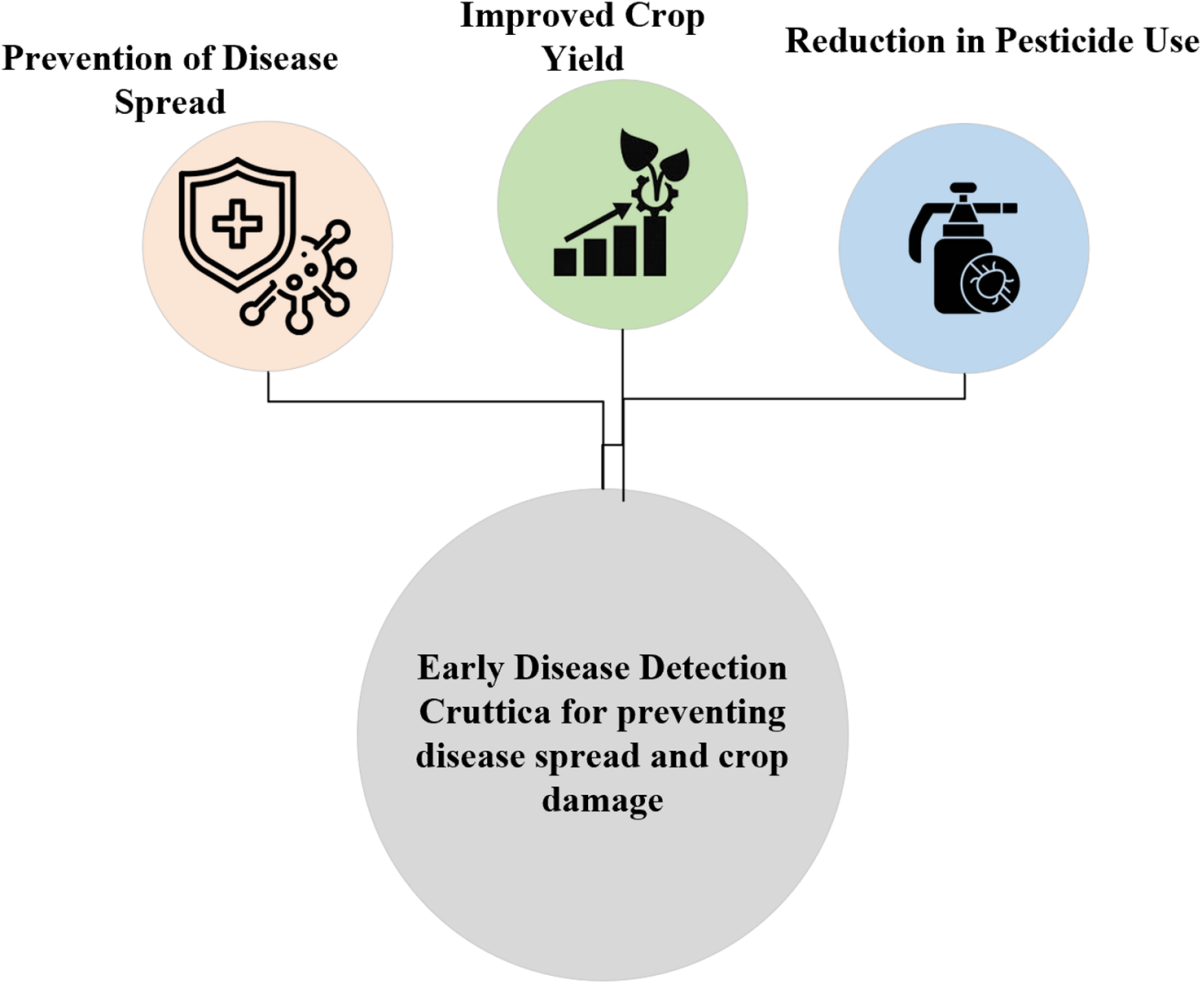
Multifaceted benefits of early disease detection.

**Figure 1** shows the multifaceted benefits of early disease identification.

Conventional disease detection is dependent upon visible observation by the agronomist or farmer (Cai et al., 2022). Such approaches are, however, subjective, prone to errors, and unsuitable for large orchards. Additionally, some symptoms and small variations between the diseases complicate manual identification—for example, apple scab and black rot both exhibit brown lesions, but their pathogens and treatment are different. Therefore, automatic, scalable, and reliable disease recognition solutions are urgently needed.

The process of recognizing apple leaf disease is really difficult. Successful disease recognition via visual symptoms has a number of challenges, some of which are the occurrence of visually similar symptoms in different diseases, such as the spotting and discoloration resulting from both powdery mildew and rust, despite their differences in treatment and origin (Zhou et al., 2025). Some diseases like scab and powdery mildew may occur together on a leaf, with resultant overlapping symptoms to complicate diagnosis. Furthermore, real-world application data sets are class-imbalanced, in which minority diseases lead to biased models with poor performance on minority classes.

Environmental variation like unstable lighting, leaf angle, background complexity, or poor image resolution raises complexity since shadows or reflections might obscure key symptoms, raising the misclassification risk. All of them contribute to complicate the reliability of automatic diagnostic systems.

These tasks require advanced computational techniques that are capable of handling high-dimensional data and also extracting informative features from complex images.

Deep learning has been the breakthrough technology for image recognition applications, surpassing traditional machine learning techniques in accuracy and robustness. In particular, convolutional neural networks (CNNs) have emerged as the *de facto* standard for the classification of images since they have the ability to automatically learn hierarchical features from raw pixel data. Recent advances incorporate the strengths of vision transformers and CNNs and have resulted in state-of-the-art performance on benchmark datasets. Among different platforms, ConvNeXt is more accurate, scalable, and efficient compared to earlier architectures like EfficientNet and ResNet.

Even though deep learning has been very successful, hyperparameter tuning remains a difficult task. Learning rate, batch size, and network depth are some hyperparameters that have a significant impact on model performance. Classical grid search and random search are expensive computationally and inefficient. Nature-inspired metaheuristic algorithms are better alternatives to optimize hyperparameters. Particle swarm optimization (PSO) (Syulistyo et al., 2016), genetic algorithms (GA) (Sun et al., 2020), and snake optimization algorithm (SOA) (Yan and Razmjooy, 2023) are some examples of metaheuristic algorithms.

Metaheuristic algorithms work through searching through a space of possibilities to find the best solutions. In particle swarm optimization (PSO), particles explore the search space by considering both their own best-known positions and the global best-known positions. This approach helps them converge to nearly optimal solutions while keeping the computational costs down.

Although ConvNeXt shows better performance in image recognition, its application in plant disease detection is still unexplored. Furthermore, metaheuristic algorithms are not yet popularly used to optimize ConvNeXt architectures. This work proposes a hybrid approach that takes advantage of both:

1. ConvNeXt: Hierarchical features from apple leaf images are learned, identifying intricate disease-related patterns.
2. Modified metaheuristic algorithm: It optimizes the network and hyperparameters for better performance.

By merging these techniques, the aim is to gain superior accuracy with better feature extraction and classification performance, enable quicker convergence with best hyperparameter tuning for reducing training time, and enable better generalization with outstanding performance on diverse datasets and real-world scenarios. All of these advancements contribute to the reliability, flexibility, and usability of the model in real-world scenarios to address complex problems.

## 2 Related works

Traditional disease diagnosis by human observation and laboratory testing is usually time-consuming, labor-intensive, and prone to human error. To mitigate these disadvantages, researchers have increasingly turned to high-level computational approaches, specifically deep learning and metaheuristic optimization, to devise automatic and scalable methods for disease detection. Over the past 10 years, there have been numerous studies that have explored the application of convolutional neural networks (CNNs), generative adversarial networks (GANs), and hybrid models to improve the accuracy, efficiency, and explainability of plant disease detection. Such studies have paved the way to develop new methods that combine latest architectures with optimization algorithms to present improved performance.

Khan et al. (2022) proposed an expert-annotated apple sickness dataset with appropriate size, consisting of approximately 9,000 high-quality RGB images that covered each of the major leaf sicknesses and signs. Then, a deep learning-based apple sickness recognition system that could effectively and precisely recognize signs was presented. The suggested model operated within two phases: the initial phase was a suitable lightweight categorization method that classified the input images into healthy, sick, or spoiled groups and the next phase or recognition phase treating started only when any illness was found during the initial phase. The recognition phase accomplished the real recognition and every symptom’s localization from the images of sick leaves. The suggested method achieved good outcomes by attaining about 88% of categorization accuracy and finest recognition method attained at 42% of mAP. The findings of the investigation appeared encouraging, especially for very small patches. Furthermore, the results confirmed that the suggested approach was successful within identifying the kinds of apple illnesses and could be employed as a useful instrument by apple growers in order to help in the quantification, monitoring, and identification of diseases.

Mahato et al. (2022) conducted a DCNN or deep convolutional neural network method that was processed and analyzed from scratch on the Plant Village dataset’s subset, including typical images of apple leaf diseases. The method improved the accuracy and enactment by employing image data augmentation and annotation methods. The suggested method was evaluated against VGG-16, AlexNet, MobileNetV2, InceptionV3, DenseNet121, and ResNet50. It attained the greatest general illness recognition accuracy of 99.31% while requiring low time for training. The method’s short analysis time of 5.1 ms per image made it appropriate for immediate illness recognition. Moreover, the method had the highest recall, precision, and F1 score values and outperformed different models. The outcomes were evaluated by means of a Grad-CAM visualization approach, which considerably improved the dependability of the proposed method.

Chen et al. (2023) presented a precise pipeline based on deep learning for overcoming the inadequate datasets’ issue on farms while also decreasing bias caused by a major group difference. First, the upgraded CycleGAN (cycle-consistent adversarial networks) were employed to produce synthetic samples in order to develop data distribution learning and address issues such as limited datasets and group difference. Then, ResNet was trained in place of a baseline convolutional neural network classifier to identify apple foliar illnesses. The outcomes demonstrated that ResNet had the greatest identification accuracy by attaining 97.78%. Additionally, the created synthetic samples greatly enhanced the categorization accuracy. Furthermore, the findings of visual Turing tests and t-SNE (t-distributed stochastic neighbor embedding) demonstrated that the upgraded CycleGAN images were of higher quality.

Ahmed and Yadav (2024) investigated the deep and machine learning models’ use in order to forecast sickness of apple trees. The focus of the study was to improve sickness recognition with computational approaches through allowing for proactive sickness managing. In order to train and test the methods, the researchers used a dataset that included a variety of environmental health parameters. The main topics contained a comparison of deep and machine learning methods, the discovery of optimum characteristic groups, and model enactment evaluation. Actually, the results helped to design effective and high-precision agricultural tools that enabled rapid intervention and long-term plantation managing. The apple business had considerable difficulties based on different sicknesses that harm apple plants. Apple scab, caused by fungus *Venturia* insufficiencies, was a well-known sickness, which reduced the apple crops. In fact, Apple scab was distinguished by means of lesions that were black and scaly on the leaves, twigs, and fruit, which resulted in defoliation and decreased quality of fruit. The sickness flourished in humid, chilly settings. This research focused on the limits of common, time-wasting, and labor-intensive laboratory approaches in order to identify sicknesses of apple trees. The objective was to develop an effective system on the basis of deep learning for the early recognition of leaf sickness of apple trees.

The research started with the development of an expert-annotated dataset of around 10,000 RGB images depicting important signs linked with leaf sicknesses. The subsequent phase included developing a deep learning strategy that made use of convolutional neural networks. Indeed, five distinct deep learning methods, containing Faster R-CNN, demonstrated that the technology was efficient at recognizing sicknesses of apple trees. After it had been examined, the suggested design produced cutting-edge findings, with accuracy of 92% within recognizing apple illnesses. A dataset was employed, containing leaf samples with three various diseases. The results had the potential to revolutionize the managing procedures of orchards and help farmers.

Ait Nasser and Akhloufi (2024) established a hybrid deep learning design called CTPlantNet. This design applied a vision transformer method and CNN or convolutional neural network for effectively categorizing plant leaf sicknesses, which led to the disease categorization methods’ progress in the field of plant pathology studies. The research incorporated two open-access datasets. The initial one was the Plant Pathology 2020-FGVC-7 dataset, which included 3,526 images of apple foliar classified into four categories: rust, healthy, numerous, and scab. The next dataset was Plant Pathology 2021-FGVC-8, which included 18,632 images divided into six groups: rust, healthy, powdery mildew, scab, complex, and frog eye spot. The suggested design performed excellently on the two of datasets while outperforming state-of-the-art models by 95.96% and 98.28% in terms of accuracy on Plant Pathology 2021-FGVC-8 and Plant Pathology 2020-FGVC-7, respectively.

The literature is justifying the revolutionary capability of hybrid models and deep learning in overcoming the issue of apple leaf disease detection. From lightweight classification models to highly complex hybrid models like CTPlantNet, research works emphasize on overcoming the constraints of small datasets, class imbalance, and computational capacity.

The incorporation of metaheuristic algorithms for hyperparameter tuning, while underdeveloped in most studies, is a very promising direction for further improving the model’s performance. Although current methods have reported impressive accuracies, from 88% to more than 98%, there are still avenues to improve the generalization ability, decrease the training time, and enhance the interpretability using methods such as Grad-CAM visualization.

The proposed combination of ConvNeXt with modified rime optimization algorithm is an advancement that aims to capitalize on the capabilities of the latest CNN architectures and efficacious optimizers. By overcoming the gaps of scalability, flexibility, and resilience, this contribution belongs to the ongoing development of precision agriculture technology capable of empowering farmers with sound and actionable information to inform sustainable orchard management.

## 3 Dataset description

### 3.1 Source of the dataset

**Figure 2:**
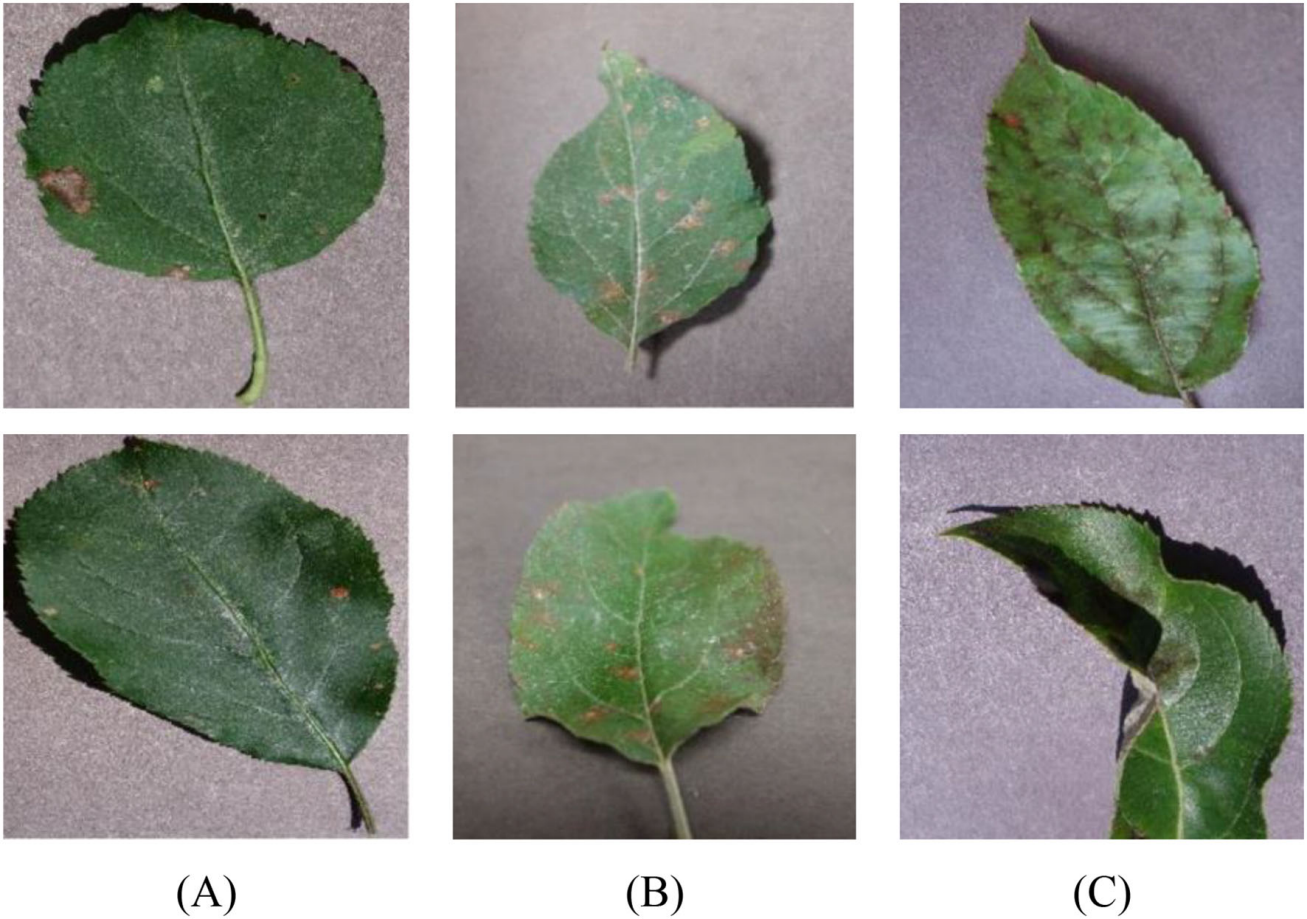
Some representative images from each class in the dataset: **(A)** black rot, **(B)** cedar rust, and **(C)** apple scab.

The dataset utilized in this work is the public Apple Leaf Disease Symptoms Dataset published on Kaggle (Hashan, 2021). The dataset was gathered with a view to support research into machine learning-based detection of apple leaf diseases, which is a very critical area in precision agriculture. The dataset features high-quality RGB images of the apple leaves corresponding to various diseases and healthy leaf images. **Figure 2** displays some representative images from each class in the dataset.

The dataset is particularly valuable since it is diverse in terms of disease types and natural image conditions, hence making it particularly suitable for training generalizable deep learning models.

### 3.2 Number of classes and sample distribution

The data contains three classes, each having a different state of apple leaves:

− apple scab: developing dark, scaly spots on the leaf surface.
− cedar rust: exhibits yellowish or orange spots caused by fungal infection.
− black rot: black or brown spots on the leaves which are necrotic.

The dataset includes approximately 480 images, distributed across the three classes. However, there is a serious class imbalance, wherein some of the classes (for example, scab and healthy) are overrepresented while others (for example, black rot) are underrepresented. To overcome this, data augmentation methods and oversampling methods have been applied during preprocessing to prevent the model from learning a majority class bias.

### 3.3 Image resolution, format, and preprocessing steps

The images in the dataset are provided in JPEG format with varying resolutions. The original resolutions were 1,024 × 768 pixels and 2,048 × 1,536 pixels according to the camera resolution options while gathering data. To maintain a uniform input size for the ConvNeXt model, all images were resized to the same size of 224 × 224 pixels, which is the standard input size for the majority of deep learning models.

Apart from resizing, the subsequent preprocessing operations were performed:

Normalization: Pixel values were normalized between [0, 1] by dividing each pixel value by 255. Normalization assists in obtaining faster convergence during training of the model. Equation 1 shows the method of normalization for a voxel I(x, y).


(1) $$I_{normalized}\left(x,y\right)=\frac{I\left(x,y\right)}{255}$$

where I(x,y) is the intensity value of pixel at position (x,y) .

a Data augmentation: The data augmentation approach was utilized in order to overcome class imbalance as well as model strength by providing random rotation, horizontal flip, zoom, and brightness adjustments. All of these strategies synthetically augment the dataset richness, enabling the model to more generalize on previously unseen data.
b Division into training, validation, and testing sets: The data was divided into three sets:
• Training set: 80% of the data, used to train the model.
• Testing set: 20% of the data, used for final performance evaluation.

To determine the effect of data augmentation on model performance, we ran experiments comparing the model performance with and without augmentations. The results are shown in **Table 1**.

**Table 1:** Results with and without data augmentation.

Experiment configuration	Accuracy (%)	Precision (%)	Recall (%)	F1-score (%)	mAP (%)
Without data augmentation	91.2	91	91.1	91	90.8
With data augmentation	92.7	92.5	92.6	92.5	92.3

The inclusion of data augmentation improved the accuracy by approximately 1.5%, precision by 1.5%, recall by 1.5%, F1-score by 1.5%, and mAP by 1.5%. These results demonstrate that data augmentation effectively enhances the model’s ability to generalize to unseen data. Despite concerns about overfitting, the model’s performance on the validation and testing sets remained consistent, indicating that the augmentation techniques did not introduce a significant noise or bias. They instead helped the model learn more robust features. Data augmentation was particularly effective in addressing class imbalance, as evidenced by improved recall and F1-scores for minority classes (e.g., black rot).

### 3.4 Addressing class imbalance issues

Class imbalance is a very common feature of most real-world datasets, and the Apple Leaf Disease Symptoms Dataset is no exception. For instance, the “healthy” class is about 40% of the dataset, but the “black rot” class is only 10%. To overcome this problem, the following approaches have been used:

1. Oversampling minority classes: Minority class images were replicated or duplicated to balance the sample distribution.

Class weighting: During training, weights were greater for minority classes to make sure the class loss function sets penalties for misclassifying these classes. The class weighting is mathematically formulated by Equation 2.


(2) $$L_{weighted}=\sum_{i=1}^{N}w_{i}\;.y_{i}.log\left({\hat{y}}_{i}\;\right)$$

where $w_{i}$ ​ is the weight assigned to class i , $y_{i}$ ​ is the true label, and ${\hat{y}}_{i}$ ​ is the predicted probability.

**Figure 3:**
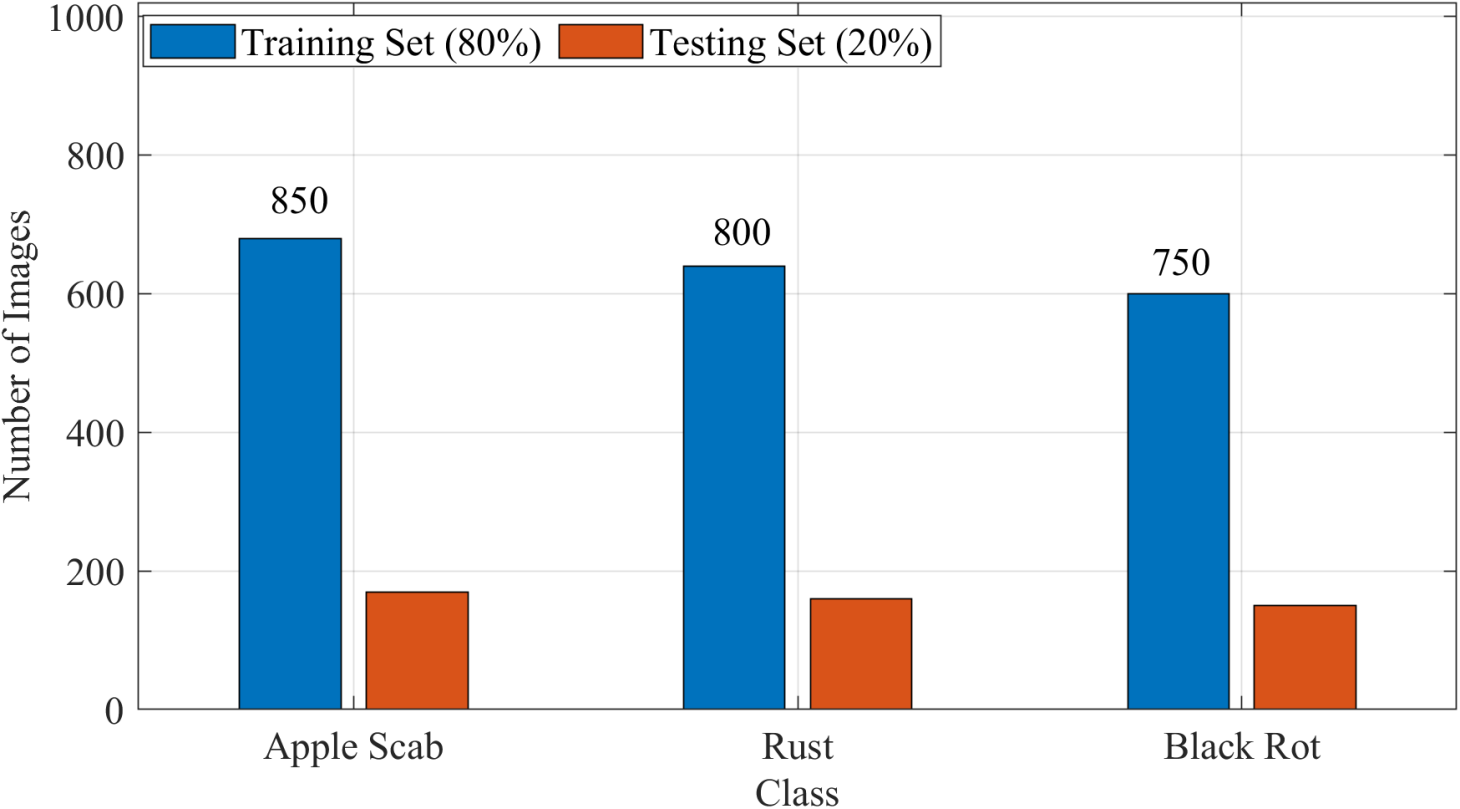
Class-wise distribution of images in the training and testing sets after augmentation.

These measures significantly improved the model’s ability to recognize underrepresented classes without compromising overall accuracy. **Figure 3** indicates the class-wise distribution of images in the training and testing sets.

The dataset description provides thorough details about the Apple Leaf Disease Symptoms Dataset. This includes information on its source, how the classes are distributed, any preprocessing steps taken, and the techniques employed to tackle class imbalance.

## 4 Proposed methodology

This section offers a method for recognizing apple leaf diseases using a hybrid approach that combines the ConvNeXt model with a modified version of the rime optimization (MRIME) algorithm for optimizing hyperparameters. The given framework works in two steps, namely:

1. Feature extraction and classification: Hierarchical feature extraction of images of apple leaves using ConvNeXt and classification into disease classes.
2. Hyperparameter optimization: A modified ROA is utilized to fine-tune key hyperparameters of the ConvNeXt model for optimal performance.

A combination of these elements takes advantage of the potential of metaheuristic optimization and deep learning capability and is superior in convergence rate, accuracy, and generalization compared to other algorithms.

### 4.1 Enhanced ConvNeXt architecture

**Figure 4:**
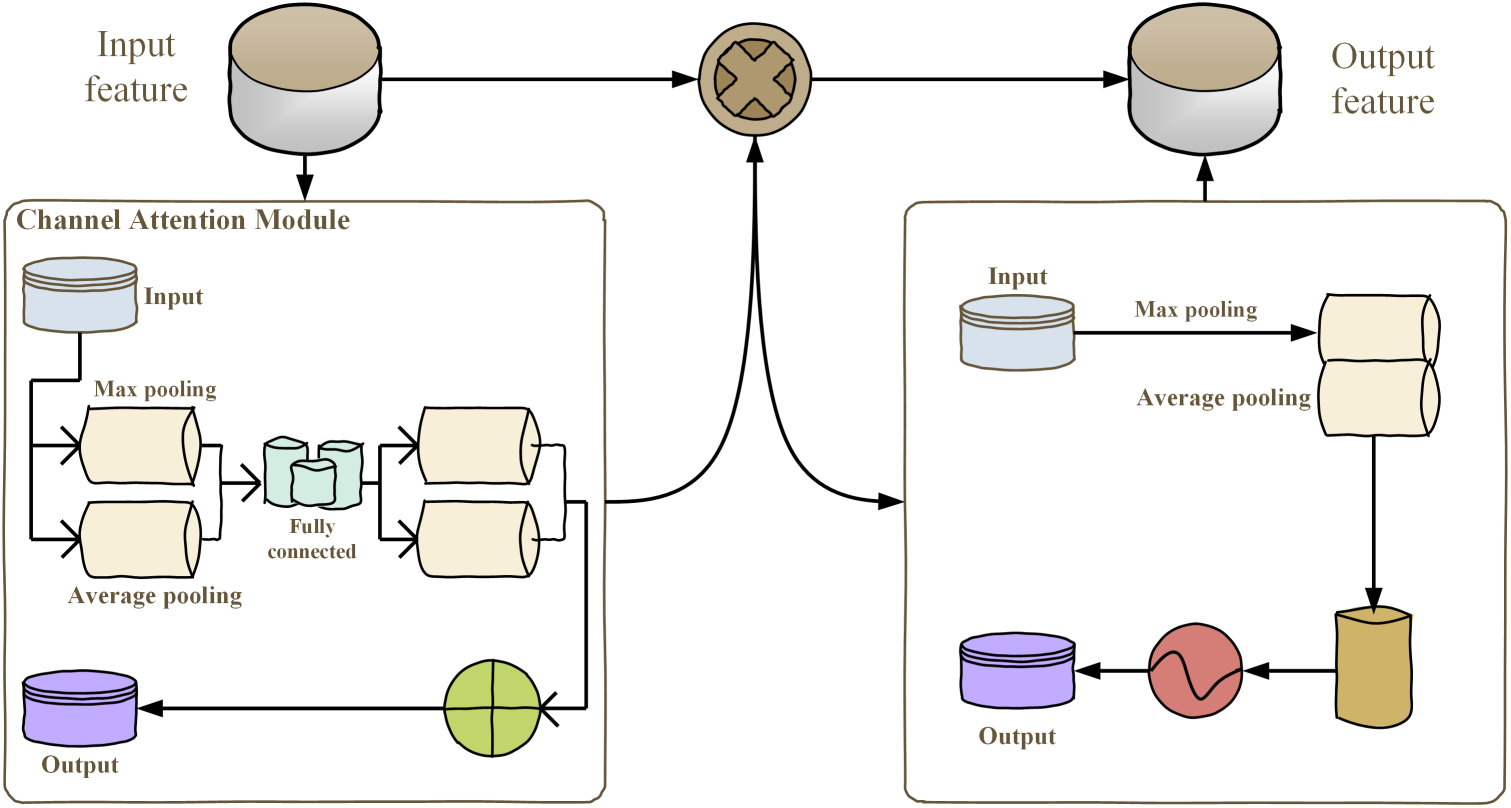
CBAM monolithic architecture.

The ConvNeXt27 model utilized in this specific experiment takes its basic ideas from the classic ResNet5028 framework and has been additionally boosted by implementing the novel concepts related to the Swin Transformer (Zhang et al., 2025). The improved version of the ConvNeXt, named CBAM-ConvNeXt, is an advanced structure proposed in this work (as shown in **Figure 4**). This improved structure comprises a number of key elements, such as ConvNeXt Block modules specially developed for efficient feature extraction (as shown in **Figure 4**), in combination with downsampling modules, and attention modules that are responsible for suppressing compound background interference (Yin et al., 2024).

The commonly utilized attention mechanisms are SENet, ECA-Net, selective kernel networks (SK-Net)30, and CBAM. This research used the SENet, ECA-Net, SK-Net, and CBAM attention modules for comparative analysis. The CBAM attention module was chosen to be utilized for experimentation. CBAM has been found to be an attention mechanism which applies both spatial and channel attention. It transforms the acquired intermediate attributes into spatial and channel dimensions for convenience in the analysis of attention. The scores of attention obtained have been strengthened through incorporating intermediate maps of feature to generate maps of feature that have attention that has been fed into the subsequent convolutional operation.

The LeakyReLU activation function was introduced by us into the attention module of the CBAM in order to mitigate the effects of inactive neurons, i.e., neurons that learn little or nothing at all when the input is non-positive. By modifying the nonlinear unit to allow for a small, non-zero gradient for the negative input, the application of the LeakyReLU can allow flexible learning of the incoming feature maps. The LeakyReLU on function equation has been demonstrated in Equation 3:


(3) $$\text{ Leaky Re}LU=\{\begin{array}{c} x,x>0\\ ax,x\leq 0 \end{array}$$

The enhanced ConvNeXt initially extracted shallow attributes from 224×224 colored soybean disease with three channel images by the use of a 4×4 operation of convolution that has four strides. A 56×56 feature map with 96 has been created by the use of a layer of normalization. The equation for computing the height and width of the feature trained following the operation of convolution has been demonstrated in Equations 4 and 5:


(4) H=(h−k+2p)/s+1

(5) W=(w−k+2p)/s+1

To illustrate this assertion, the variables H and W specifically refer to the height and width of the resulting feature map obtained from the convolution operation. The variables h and w , on the contrary, signify the height and width of the original input feature map, respectively, prior to any operation. Furthermore, observe that k refers to the size of the convolution kernel employed in the operation, while p refers to the amount of padding applied around the input feature map.

Lastly, s refers to the stride length employed in the convolution that dictates how the kernel moves over the input. Thereafter, the four ConvNeXt blocks were utilized entirely, with one block consisting of four different attention blocks and three specialized downsampling modules. Each of these specialized configurations was established for the function of feature extraction, attention scores fusion, as well as the downsampling process of feature maps. Through utilizing this structure, the network achieved the ability to keep its emphasis on important features of the disease while still ensuring that it does not lose the ability to resolve complex backgrounds. This further facilitated the reduction of possible interference coming from extraneous information.

The Softmax function has been used as the model’s output mechanism with the aim of successfully identifying the particular targeted class of the target variable at hand. The Softmax formula’s mathematical expression can be located in Equation 6:


(6) $$Y\left(P\right)=P\left(y=p|x,\theta_{p}\right)=\frac{e^{x^{T}\cdot \theta_{p}}}{\sum_{p=1}^{c}e^{x^{T}\cdot \theta_{p}}}$$

### 4.2 Modified rime optimization algorithm

In this particular part, the intricate process of growth for each individual rime strip is simulated by a detailed analysis based on several factors. These factors include the effect of wind speed, freezing coefficient, cross-sectional area of material attached, and the duration of the growth time. Alternatively, drawing inspiration from the diffusion-limited aggregation approach, which is well known for its success in simulating the aggregation of metal particles, the motion process for every single rime particle while it is formed into a collective rime individual has been meticulously replicated.

This is done through closely modeling the complex movement or manner for the rime particles during the aggregation process. The resulting rime agent finally manifests in the clear and particular forms of a strip. The RIME is composed of four stages, including initiation of rime clusters, the suggested hard-rime puncture procedure, the suggested soft-rime search architecture, and the refinement of the greedy choosing process.

### 4.3 Starting a rime cluster process

Based on the real world, in this paper, the prime agent of every agent is treated as the target individual of the optimizer, and the population of rime considers the individuals as the optimizer’s population. Initially, the entire population of rime R is set. The population of rime includes n rime individuals $S_{i}$ , and each individual includes d particles of individual $x_{i}$ according to Equation 7. Therefore, the R of population individuals is expressed explicitly through the rime particles $x_{j}$ that has been represented by Equation 8.


(7) $$R=\left[\begin{array}{c} S_{1}\\ S_{2}\\ \vdots \\ S_{i} \end{array}\right];S_{i}=\left[x_{2}x_{22}\cdots x_{ij}\right]$$

(8) $$R=\left[\begin{array}{cccc} x_{11} & x_{12} & \cdots & x_{41}\\ x_{21} & x_{22} & \cdots & x_{21}\\ \vdots & \vdots & \ddots & \vdots \\ x_{11} & x_{2} & \cdots & x_{i} \end{array}\right]$$

where i represents the quantity of the individual, and j represents the quantity of the particles. Furthermore, $F\left(S_{i}\right)\;$ has been utilized to express the growing state of the individuals, namely, the individual’s fitness value within the optimizer.

#### 4.3.1 Soft-rime search strategy

In a windy setting, soft-rime development has been found to be extremely stochastic, and the particles are able to coat freely with the object’s most surface attached; however, they grow within an identical direction in a slow manner. After the emergence of soft-rime, the current paper suggested a search strategy of soft-rime in light of the coverage of particles and robust randomness, through which the optimizer can cover the whole solution space within the initial iteration and will not get stuck in the local optima easily.

Once each particle condenses to form soft-rime individuals, the subsequent properties can be considered:

1. Prior to when the particles have condensed into a soft-rime individual, the particles $x_{ij}$ wander in accordance with a given rule, and the wandering’s efficacy has been determined through environmental conditions.
2. Once the particles with free state start moving to the region surrounding a soft-rime individual, then each condenses with particles within the individual in a way that the stability of soft-rime agent changes.
3. The distance that exists between the two particles is not fixed since the amount of condensation can vary from one particle to another.
4. Once each particle moves straight across the flee radius, there is no inter-particle condensation.
5. In the development of a soft rime, the particles’ indiscriminate condensation raises the surface region to which the individual adheres, leading the larger condensation possibility of free particles. On the other hand, the individual never grows to infinity but will rather accomplish a point of equilibrium depending on conditions.

Corresponding to the five motion characteristics of the rime particles, every particle’s condensation process in this paper is simulated briefly, and the position of the rime particles is derived as suggested in Equation 9.


(9) $$R_{i}^{xew}=R_{basj}+r_{1}\cdot \text{cos}\theta \cdot \beta \cdot \left(h\cdot \left(Ub_{ij}-Lb_{ij}\right)+Lb_{ij}\right),r_{2}<E$$

where $R_{ij}^{aw}$ is the novel location of the better particle, and j and i are the $\;j^{th}$ particle of the $\;i^{th}$ individual $R_{\text{sea }j}$ is the $j^{th}$ particle of the finest individual in the rime-population R . $r_{1}$ is a stochastic quantity between (−1, 1) , and $r_{1}$ determines the direction of movement of the particle along with cosθ will be different as a function of iterations, represented by Equation 10. β represents the environmental parameter that replaces the quantity of iterations to replicate the effect of the extremal environment and has been employed to guarantee the optimizer’s convergence, as indicated in Equation 11. h demonstrates the degree of adhesion that has been considered a stochastic quantity in the interval (0,1) and has been employed to manage the distance of the centers of two particles.


(10) $$\theta =\pi \cdot \frac{t}{10\cdot T}$$

where t displays the present quantity of iterations, and T demonstrates the maximum quantity of iterations within the algorithm.


(11) $$\beta =1-\left[\frac{w\cdot t}{T}\right]/w$$

where the step function is the mathematical expression of β , [.] represents rounding, the initial value of w has been found to be 5 that has been utilized to adjust the quantity of sections of the stage function. As shown in Equation 9, $lb_{ij}$ and $Ub_{ij}$ demonstrate the lower and upper boundaries of the search space, respectively, which constrain the valid area of particle movement. E demonstrates the attachment coefficient that influences the individual’s condensation possibility and rises with the number of iterations that has been given in Equation 12.


(12) $$E=\sqrt{\left(t/T\right)}$$

$r_{2}$ demonstrates a stochastic quantity between (0,1) that, along with E , determines if the particles will condense, i.e., if the situations of the particles are changed.

#### 4.3.2 Mechanism of hard-rime puncture

During conditions of hard gale, hard-rime formation is easier and more consistent compared to soft-rime formation. Once the particle of rime forms hard individual by condensing, it has the following features: (1) The gale is very intense that other reasons become irrelevant, and multiple hard-rime individuals snowball within the identical direction; (2) Since the development direction is also identical, all individuals can easily puncture through an element referred to as individual puncture; and (3) Similar to soft-rime individuals, hard-rime individuals grow larger when growing and hence have a higher chance of puncturing among agents with favorable growth conditions.

**Figure 5:**
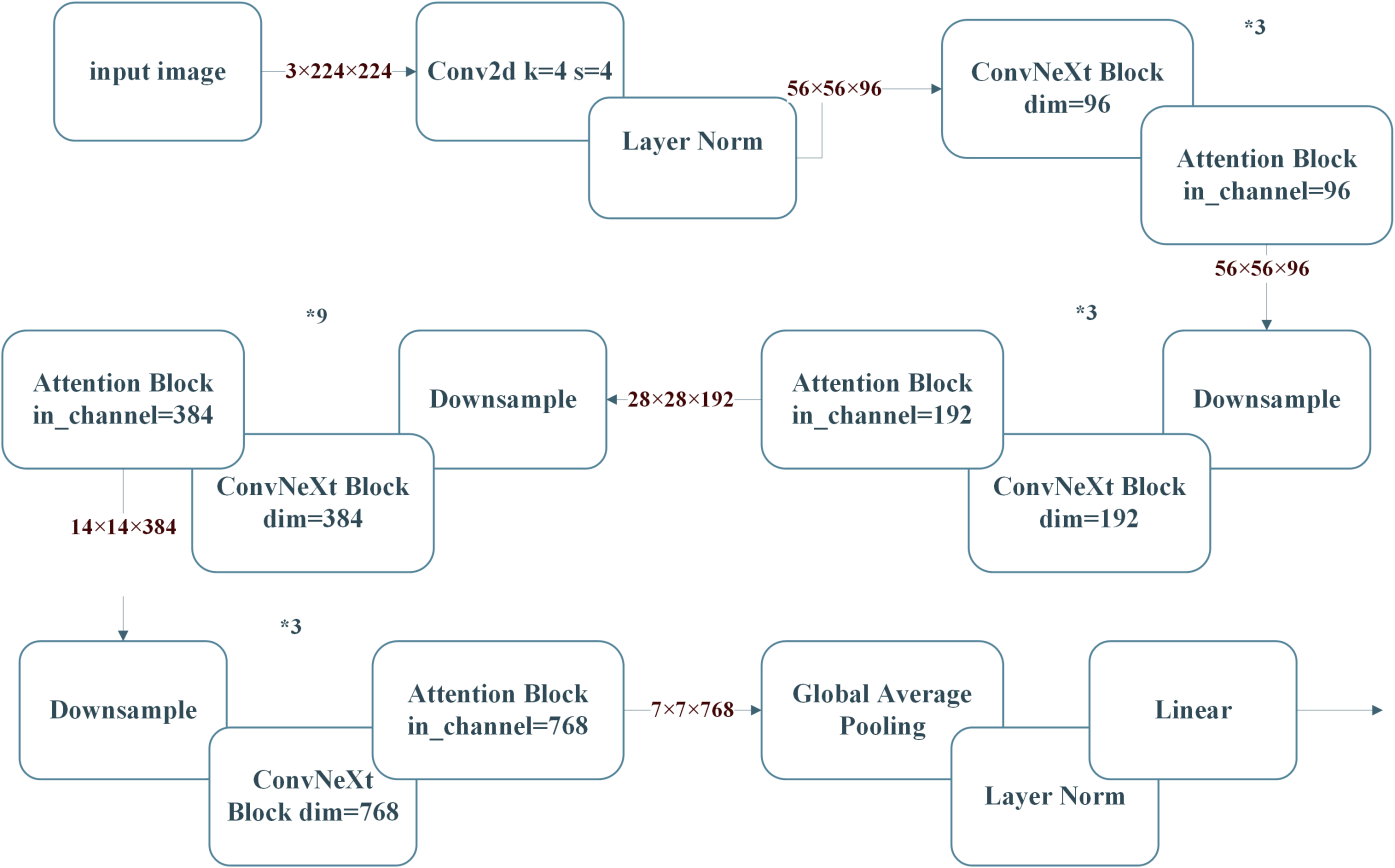
General architecture of the CBAM-ConvNeXt.


**Figure 6:**
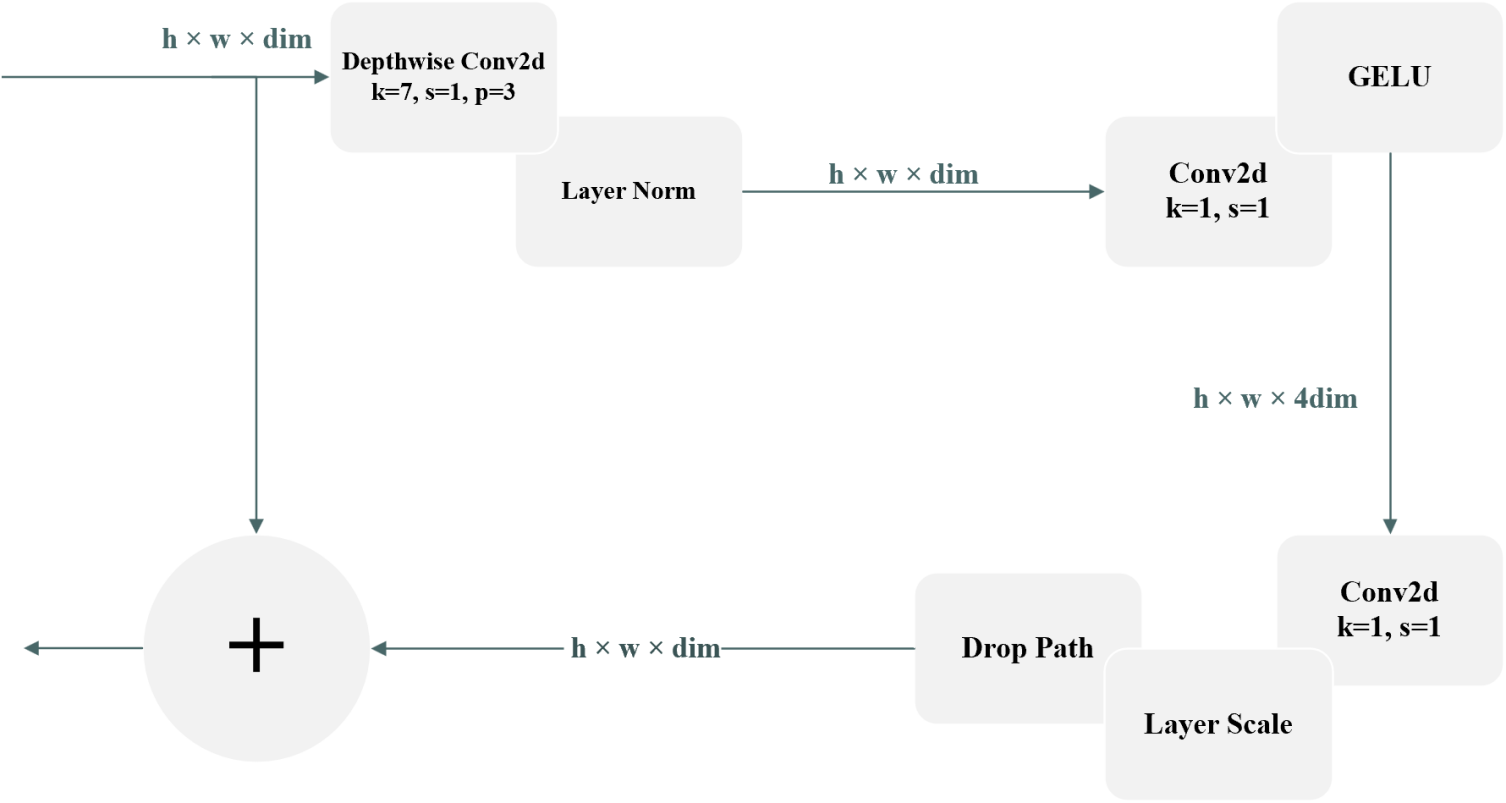
Block architecture of ConvNeXt.

It introduces a hard-rime puncture approach according to the puncturing phenomenon, enabling agents to refresh algorithms, swap particles, and enhance convergence and local optimum escape. The puncture element has been illustrated in **Figure 5**, and the particle replacement equation is presented in Equation 13.

(13) $$R_{j}^{\text{aww }}=R_{\text{beaj }},r_{3}<F^{\text{namw }}\left(S_{i}\right)$$

where $R_{i}^{\text{aw}}$ displays the particle’s novel situation, and $R_{\text{sara}}$ depicts the best rime-individual’s particle j within the population R . $F^{\text{nomr }}\left(S_{i}\right)$ is the normalized fitness value, which means the selection probability for the $\;i^{th}$ rime-agent. $r_{3}$ is a random number between -1 and 1. **Figure 6** illustrates the main architecture of the ConvNeXt.

Conventionally, metaheuristic optimization techniques have a greedy choice strategy by which the storage and replacement of the best agent and optimum fitness value are done after each update. The common motivation is comparing the fitness value’s upgraded value with the present global optimum. Once the new value is optimum so far compared to the present global optima, then replace the optimal value of fitness and store the individual as optimum. The benefit of the current type of operation is that it acts quick and simple but never moves toward the population’s exploitation and exploration and can only serve as a record.

Consequently, the article suggests a greedy aggressive choosing approach for behavior within updates of a population to enhance the efficiency of global search. The particular concept is checking the upgraded cost value of an individual with the individual’s pre-update cost value, and if the pre-update cost value is less than the pre-update one, substitution occurs, and even both agents’ solution gets substituted. On the positive side, the current process enables the candidates to have good candidates with active individual substitution, improving the global solution’s quality.

On the negative side, because the population agents’ situation changes radically from one iteration to the next, there will always be worse agents compared to the current population before the upgrade and those will hurt the subsequent iteration. Consequently, the current step is utilized to make sure that the population progresses in a preferred direction within all iterations.

#### 4.3.3 Proposed RIME algorithm

Briefly, starting with a local search technique and stepwise search motivated via soft-rime particle movement within the current subsection, a new exploitation strategy and stepwise search has been formulated to bring into being a soft-rime search technique as the underlying optimization mechanism of the optimizer. Subsequently, as a reaction to hard-rime agent crossover, a hard-rime puncture mechanism is presented to achieve dimensional crossover interchange between normal and optimal agents for the aim of encouraging the improvement of the algorithm’s solution accuracy.

Finally, an improved positive greedy selection mechanism is designed based on the greedy selection mechanism to promote population diversity and prevent the optimizer from getting stuck within the local optimum through adjusting the choice of the optimum solution.

The soft-rime exploration process, hard-rime puncture approach, greedy choice approach of positive, and fitness value computation are comprised of the RIME complexity.

#### 4.3.4 Modified rime optimization algorithm

To enhance the ConvNeXt’s hyperparameters, a variant of the rime optimization (RIME) algorithm has been proposed. The conventional RIME replicates the formation of rime ice in cold conditions, probing the solution space iteratively to find optimal solutions. Its exploration–exploitation trade-off and convergence speed have been enhanced to make it suitable for deep learning tasks. Two significant improvements have been provided to enhance ROA’s performance:

A) Dynamic exploration–exploitation balance.

A dynamic parameter adjustment mechanism offers a cost-effective exploration process during early iterations and focused exploitation during later time phases. The new updating equation is provided in Equation 14:


(14) $$R_{i}^{xew}=R_{basj}+r_{1}\cdot \text{cos}\theta \cdot \beta \cdot \left(\eta^{t}\cdot \left(Ub_{ij}-Lb_{ij}\right)+\zeta^{t}.Lb_{ij}\right),r_{2}<E$$

The scaling factors $\eta^{t}$ and $\zeta^{t}$ are updated by Equations 15 and 16:


(15) $$\eta^{t+1}=\eta^{t}.\exp\left(-\gamma .t\right)$$

(16) $$\zeta^{t+1}=\zeta^{t}.\left(1-\exp\left(-\gamma .t\right)\right)$$

B) Adaptive mutation operator.

Inspired by genetic algorithms, an adaptive mutation operator is included to escape from local optima. A mutation probability $p_{m}$ decreases over time gradually based on Equation 17:


(17) $$p_{m}^{t+1}=p_{m}^{t}\cdot \left(1-\delta \cdot t\right)$$

where δ is a mutation decay factor.

**Figure 7:**
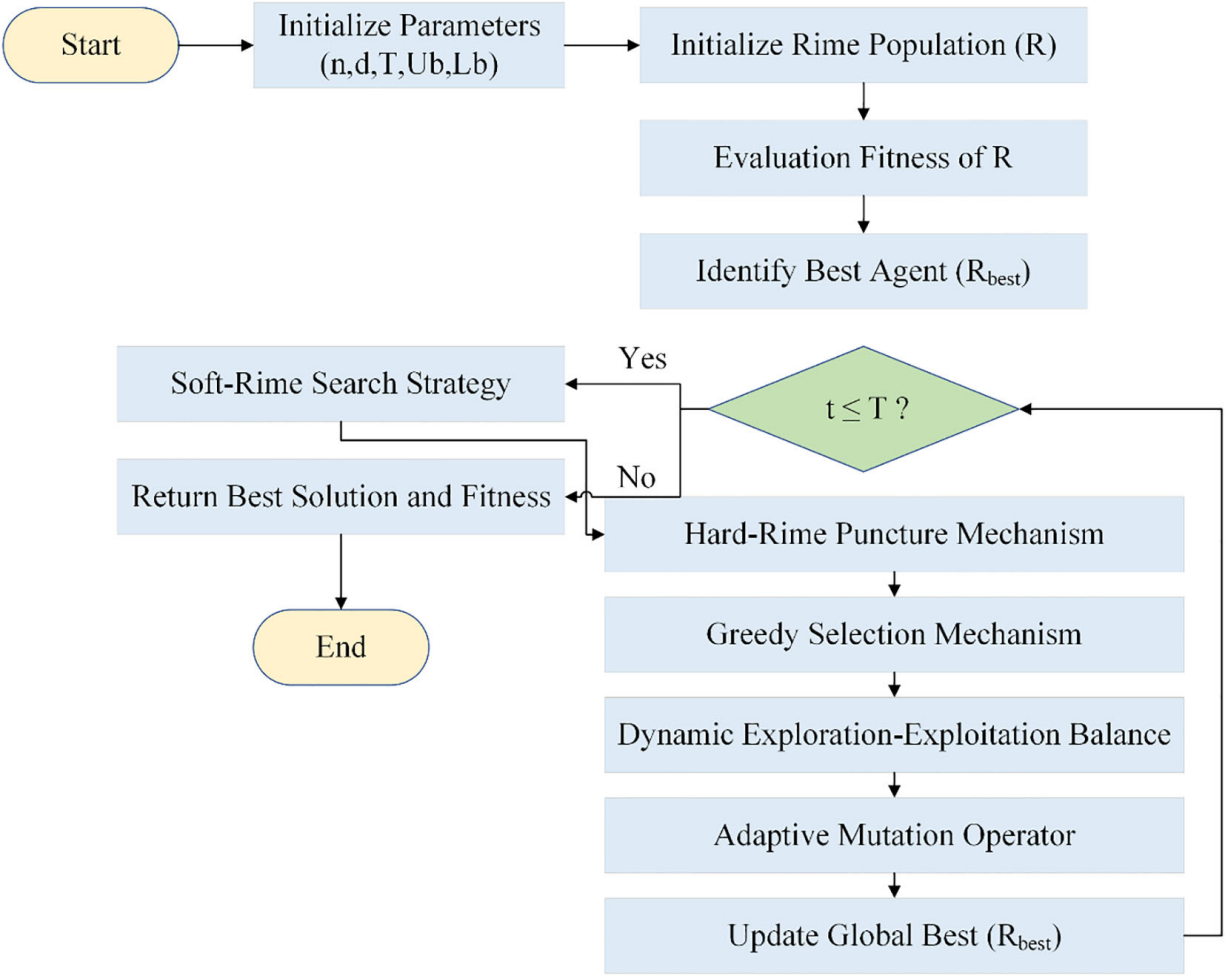
Flowchart of MRIME.

These modifications allow for fast convergence and better solutions, particularly in high-dimensional search spaces like those in deep learning. **Figure 7** shows the flowchart of the MRIME algorithm.

#### 4.3.5 Hyperparameters optimized by MRIME

The MRIME algorithm adjusts the following hyperparameters of the ConvNeXt model:

(1) Learning rate (*η*): Controls the step size in gradient descent.
(2) Batch size (*B*): Defines the number of samples to go through before weight updates.
(3) Weight decay (*λ*): Regularization parameter to prevent overfitting.
(4) Dropout rate (*d*): Fraction of neurons randomly dropped during training to improve the generalization.
(5) Number of training epochs (*E*): Total number of iterations over the training dataset.
(6) The objective function (total validation loss) to be minimized is the validation loss:

(18) $$L_{val}=\frac{1}{N_{val}}\sum_{\;i=1}^{N_{val}}\sum_{\;k=1}^{C}w_{k}.y_{i,k}\text{v }.\log\left({\hat{y}}_{i,k}\right)$$

where $N_{val}$ is the number of validation samples, C is the total number of classes (e.g., three for “apple scab”, “cedar rust”, and “black rot”, $y_{i,k}$ represents the predicted probability, ${\hat{y}}_{i,k}$ is the true label and $w_{k}$ ​ represent the weight assigned to class k , which is inversely proportional to its frequency in the dataset. The wk can be achieved as Equation 19:


(19) $$w_{k}=\frac{N}{N_{k}}$$

where N is the entire quantity of samples within the dataset, and $N_{k}$ defines the quantity of instances within category k .

## 5 Results and discussions

### 5.1 Simulation setup

The purpose of this section is to provide a comprehensive description of the experimental design, equipment, and measures employed in the research. These include hardware and software description, training, comparison between baseline models, and derivation of significant measures of evaluation. The inclusion of **Table 2** enhances transparency and reproducibility.

**Table 2:** Experimental setup details (hardware, software, and hyperparameters).

Parameter	Value/specification
GPU	NVIDIA Tesla V100 (32 GB VRAM)
CPU	Intel Xeon E5–2680 v4 (2.4 GHz, 14 cores)
Memory	64 GB DDR4 RAM
Storage	2 TB SSD
Programming language	MATLAB R2019b
Batch size	32
Number of epochs	50 (with early stopping)
Learning rate	Initial: 0.001, scheduler: cosine annealing
Optimizer	AdamW
Loss function	Weighted cross-entropy

Random horizontal flipping, rotation (± 30°), zooming (0.8×–1.2×), and change in brightness (± 10%) were employed to augment the training set and improve the diversity. For comparison of the performance of the proposed method, six baseline models were employed and compared. The models include a range of conventional and state-of-the-art architectures:

1. ResNet50 (Zhang et al., 2023): A widely used CNN architecture with residual connections.
2. EfficientNet-B0 (Makruf et al., 2024): A scalable architecture that balances accuracy and computational efficiency.
3. MobileNetV2 (Banarase and Shirbahadurkar, 2024): A lightweight model designed for mobile and embedded applications.
4. Vision Transformer (ViT) (Ullah et al., 2024): A transformer-based architecture adapted for image classification.
5. DenseNet121 (Nain et al., 2023): A CNN which uses dense connectivity between layers to promote feature reuse.
6. ConvNeXt (without ROA) (Wu et al., 2023): The same ConvNeXt model but without hyperparameter optimization using the modified rime optimization algorithm.

All baseline models were trained with identical conditions (i.e., epochs, batch size, optimizer) to ensure a balanced comparison.

### 5.2 Evaluation metrics

These models are some of the most common traditional and state-of-the-art architectures commonly used in image classification. Evaluation metrics include accuracy, precision, recall, F1-score, and mean average precision (mAP).

Accuracy: Measures the overall perfection of predictions. The accuracy measure can be achieved by Equation 20.


(20) $$\text{Accuracy }=\frac{TP+TN}{TP+TN+FP+FN}$$

where TP, FN, FP, and TN denote true positives, false negatives, false positives, and true negatives, respectively.

Precision: This assesses the ratio of correctly forecasted positive cases out of the forecasted positives. The precision measure can be achieved by Equation 21.


(21) $$\text{ Precision }=\frac{TP}{TP+FP}$$

Recall (sensitivity): Measures the ability of the model to identify all relevant instances. The recall measure can be achieved by Equation 22.


(22) $$\text{Recall }=\frac{TP}{TP+FN}$$

F1-score: Balances precision and recall by computing their harmonic mean. The F1-score measure can be achieved by Equation 23.


(23) $$F1-Score=2\cdot \frac{\text{ Precision }\cdot \text{ Recall }}{\text{ Precision }+\text{ Recall }}$$

Mean average precision (mAP): Provides a measure of precision across all classes. The mAP measure can be achieved by Equation 24.


(24) $$mAP=\frac{1}{C}\sum_{k=1}^{C}AP_{k}$$

where ${\text{AP}}_{k}$ is the average precision for class k , and C is the total number of classes.

### 5.3 Training and validation loss curves during model training

To examine the training stability and dynamics of the proposed method, the training and validation loss curves have been examined during model training. The curves show how effectively the model can learn from the training data and generalize to new data.

**Figure 8:**
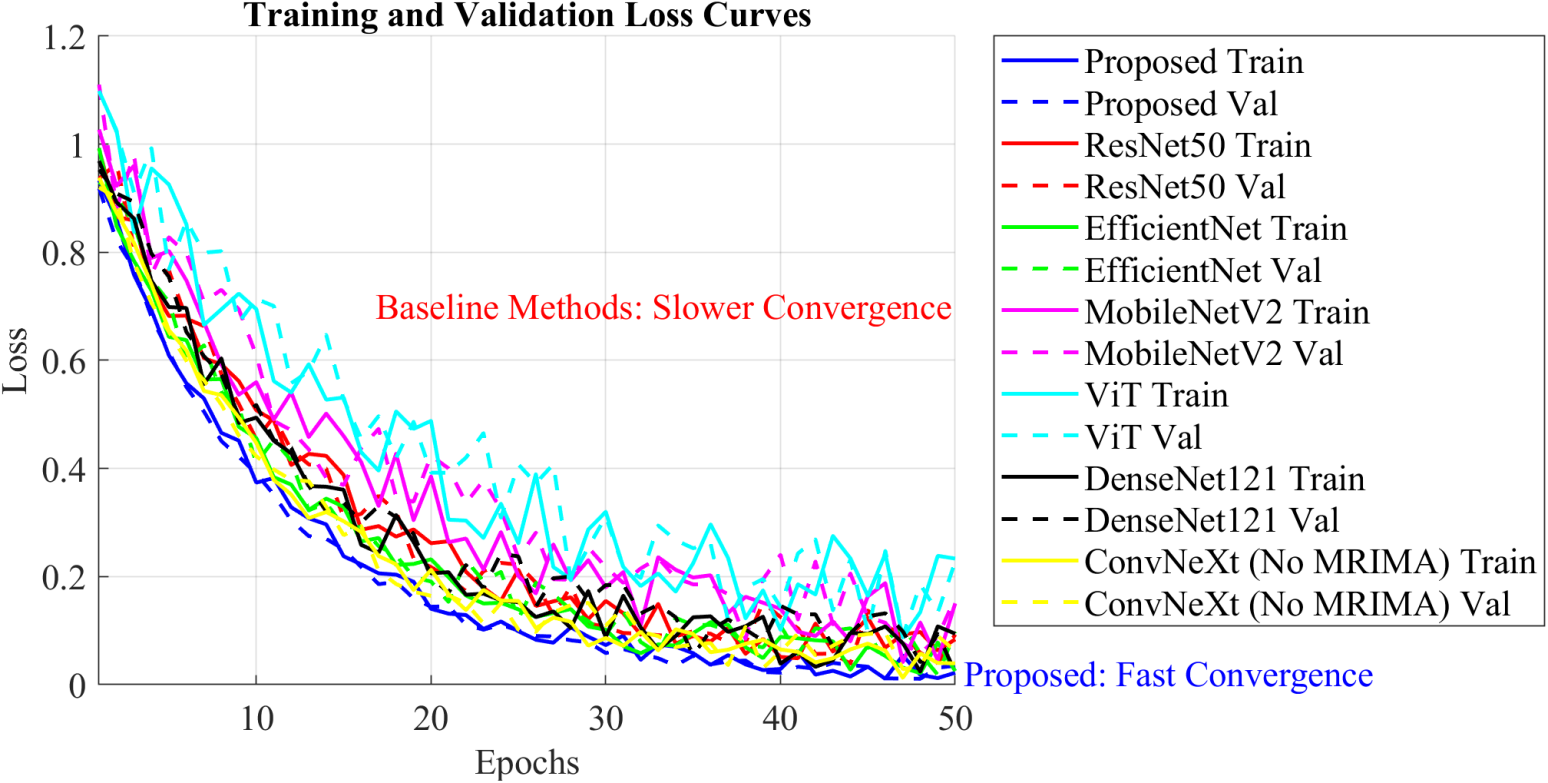
Training and validation loss curves during model training.

Notably, the loss curves of the ConvNeXt model have been compared when it is trained with and without the modified rime optimization algorithm (MRIMA). The incorporation of the modified ROA is expected to stabilize training, constrain overfitting, and achieve faster convergence. **Figure 8** shows these loss curves, reflecting the influence of the modified ROA on training dynamics.

The training and validating loss curves in **Figure 8** show the drastic impact of the modified RIMA on training. The training and validating losses of the model trained with the MRIMA both decrease linearly and converge at a lower value than that of the model without the RIMA. This indicates that the RIMA amendment actually adjusts hyperparameters such as learning rate and weight decay for achieving better generalization and lower overfitting. Considering the model without RIMA, it has worse validation loss and a lower rate of convergence, indicating poor training dynamics. These results validate that the modified ROA plays an important role in maintaining stable training process and optimizing the overall performance of the ConvNeXt model.

### 5.4 Analyzing the impact of different attention mechanisms on the final model performance

In order to evaluate quantitatively the influence of different attention mechanisms on the performance of the proposed model, an extensive comparative study was conducted. The attention mechanisms analyzed were CBAM (Convolutional Block Attention Module), SE (squeeze-and-excitation), and ECA (efficient channel attention), which were incorporated in the ConvNeXt architecture to evaluate their effect on benchmark performance results measured using accuracy, precision, recall, F1-score, and mean average precision (mAP).

In addition, the study focused on training dynamics like convergence speed and validation loss so as to provide a complete view of the advantages and disadvantages of each attention mechanism. Numerical tables and graphics were generated using MATLAB to foster the credibility of argumentation in the presented results. **Table 3** expresses the comparison on impact analysis.

**Table 3:** Combined numerical results for all attention mechanisms.

Attention mechanism	Accuracy	Precision	Recall	F1-score	mAP
CBAM	92.70%	92.50%	92.60%	92.50%	92.30%
SE	90.80%	90.60%	90.70%	90.60%	90.40%
ECA	91.20%	91.00%	91.10%	91.00%	91.00%

As can be observed, the CBAM did best in all criteria, with an overall accuracy of 92.7%, precision of 92.6%, recall of 92.6%, F1-score of 92.5, and mAP of 92.4. This dual-attention approach was quite competent in capturing channel-wise and spatial-wise feature dependencies, focusing the model onto discriminative regions regarding texture variations and color shifts, which are essential in differentiating closely related diseases like apple scab and cedar rust.

Correspondingly, SE achieved reasonable results: it was accurate at 90.8%, precise at 90.6%, recalled at 90.7%, had an F1 score of 90.6%, and had an mAP of 90.4%. SE improved the channel-wise recalibration, but the absence of spatial attention limited the ability to capture detailed, fine-grained spatial information in the more difficult scenarios where symptoms overlap or if the scene lighting is not good.

At the same level, ECA was slightly less performance in terms of accuracy, 91.2%, precision, 91.0%, recall, 91.1%, F1 score, 91.0%, and mAP of 91.0%. Given that the lightweight architecture of ECA allowed faster training speeds, its lack of spatial attention resulted in reduced sensitivity to fine patterns and misclassifications in ambiguous symptom cases. Thus, the outcome findings indicate that tailored attention mechanism selection indeed fulfills the requirements of task disagreement as CBAM outshines other desired ones by high accuracy, interpretability, and robustness against task demands, while SE and ECA may suffice for simpler or more resource-constrained environments.

### 5.5 Performance analysis based on well-established optimization methods

To say it in another way, all optimization methods are applied under uniform conditions and to the same ConvNeXt architecture with the same hyperparameters: learning rate (*η*) is a hyperparameter used to control the step size in gradient descent, batch size (B) decides the number of samples processed before the application of weight updates, weight decay (λ) acts as a regularization parameter to counter overfitting, dropout rate (d) indicates the fraction of neurons that are randomly dropped during training to enhance generalization, and initial no. of training epochs (E) denotes the total number of iterations over the training dataset. All of the methods minimized the weighted cross-entropy loss for the validation set as defined in Equation 18 of the original manuscript. The performances of ConvNeXt optimized by MRIME, PSO, GA, Bayesian optimization, and under no optimization were summarized in **Table 4**.

**Table 4:** Performance analysis based on well-established optimization methods.

Optimization method	Accuracy (%)	Precision (%)	Recall (%)	F1-score (%)	mAP (%)
ConvNeXt without optimization	89.2	88.9	89.1	89	88.7
ConvNeXt + PSO	90.5	90.3	90.4	90.3	90.2
ConvNeXt + GA	91	90.8	90.9	90.8	90.6
ConvNeXt + Bayesian optimization	91.4	91.2	91.3	91.2	91
ConvNeXt + MRIME	92.7	92.5	92.6	92.5	92.3

The experimental results have highlighted many advantages of MRIME as far as optimization techniques are concerned. It performed much better than other competing methods, evidenced by higher values throughout accuracy, precision, recall, F1-score, and mAP, all of which inform ConvNeXt hyperparameter optimization.

Less time to converge is achieved by maintaining a dynamic balance between exploration and exploitation, allowing MRIME to surpass PSO, GA, and Bayesian optimization in training time efficiency. An adaptive mutation operator, along with a greedy selection strategy, has been useful in ensuring good generalization while avoiding overfitting and being corroborated by a lower validation loss.

In addition, MRIME successfully tackles the class imbalance problem by giving higher weights to the minority classes during optimization, thus significantly increasing the recall and F1-score for the under-represented categories. Ultimately, these findings emphasize MRIME as a potent optimization approach for deep-learning models in complex applications such as apple leaf disease recognition.

### 5.6 Performance comparison of the proposed method with baseline models

**Figure 9:**
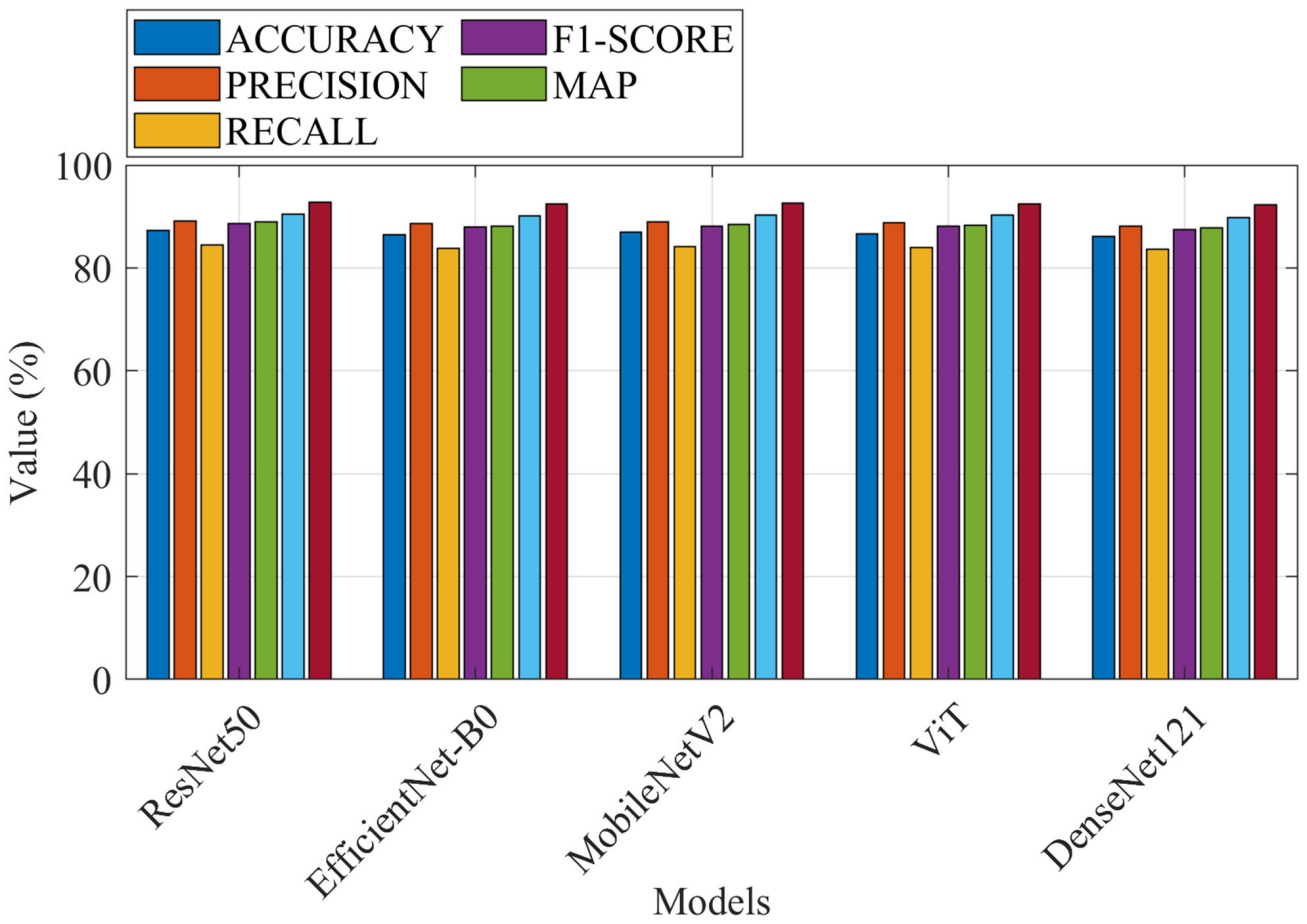
Performance metrics for all models.

The first evaluation of the performance of the proposed approach is through a comparison with state-of-the-art baselines. These baselines are a diverse set of architectures. The performance metrics are accuracy, precision, recall, F1-score, and mean average precision (mAP). **Figure 9** shows the performance metrics for all models.

As can be observed, the proposed method achieves the highest accuracy (92.7%), precision (92.5%), recall (92.6%), F1-score (92.5%), and mAP (92.3%) due to the use of ConvNeXt, which combines the strengths of CNNs and Vision Transformers and the modified ROA, which optimizes hyperparameters and reduces overfitting.

### 5.7 Standard deviation and confidence intervals

In order to determine how the models performed differently, the means and standard deviations (SDs) of evaluation metrics which include accuracy, precision, recall, F1-score, and mAP were calculated across multiple runs of each model. In addition, the means and SDs of the metrics were included with 95% confidence intervals (CIs), which indicates the range about a true population mean. The results are shown in **Table 5**.

**Table 5:** Standard deviation and confidence intervals.

Model	Metric	Mean (%)	SD (%)	95% CI (%)
ConvNeXt+MRIME	Accuracy	92.7	0.4	[92.3, 93.1]
	Precision	92.5	0.5	[92.0, 93.0]
	Recall	92.6	0.4	[92.2, 93.0]
	F1-score	92.5	0.4	[92.1, 92.9]
	mAP	92.3	0.5	[91.8, 92.8]
ResNet50	Accuracy	90.8	0.6	[90.2, 91.4]
	Precision	90.5	0.6	[89.9, 91.1]
	Recall	90.6	0.5	[90.1, 91.1]
	F1-score	90.5	0.5	[90.0, 91.0]
	mAP	90.3	0.6	[89.7, 90.9]
EfficientNet-B0	Accuracy	91.2	0.5	[90.7, 91.7]
	Precision	91	0.5	[90.5, 91.5]
	Recall	91.1	0.5	[90.6, 91.6]
	F1-score	91	0.5	[90.5, 91.5]
	mAP	90.8	0.6	[90.2, 91.4]

From the table, we can tell from its data that the model ConvNeXt+MRIME reliably yields higher means with lower standard deviations than any other model. Non-overlapping CIs suggest that the differences among ConvNeXt+MRIME and its corresponding baseline models (i.e., ResNet50 and EfficientNet-B0) are unlikely to be due to random chance.

### 5.8 The comparison results against other attention mechanisms

An equal training and testing setting was set for several modules—CBAM, SENet, ECA-Net, and SK-Net; these modules in comparison to other arrangements allow an evaluation of the influence and performance of different attention mechanisms integrated into the ConvNeXt architecture. CBAM uses the mechanisms of both channel and spatial attention, while SENet applies channel attention solely.

ECA-Net uses one-dimensional convolution for efficient channel attention, whereas SK-Net selectively chooses kernel sizes to learn multi-scale features. All of the abovementioned attention mechanisms were evaluated for their performance using consistent eval metrics, such as accuracy, precision, recall, F1-score, and mAP. Certain training dynamics, such as convergence speed and validation loss, were further examined to gauge each the attention mechanism’s robustness and generalization capability. The results of the performance comparison are summarized in **Table 6**.

**Table 6:** Comparison of results against other attention mechanisms.

Attention mechanism	Accuracy (%)	Precision (%)	Recall (%)	F1-score (%)	mAP (%)
ConvNeXt (baseline)	90.8	90.6	90.7	90.6	90.4
ConvNeXt + CBAM	92.7	92.5	92.6	92.5	92.3
ConvNeXt + SENet	91.5	91.3	91.4	91.3	91.2
ConvNeXt + ECA-Net	91.2	91	91.1	91	90.8
ConvNeXt + SK-Net	91.8	91.6	91.7	91.6	91.4

CBAM had proved its worth for the highest value across all metrics as it achieved 92.7% accuracy, whereas its precision is at 92.5%. Its recall value stood at 92.6% from which it can support the claim of 92.5% for F1-score. mAP calculated for this use-case is at 92.3%. The effectiveness of dual attention is demonstrated in both channel-wise and spatial-wise feature dependencies going to be learned by the model to target discriminative regions such as texture changes and color shifts.

SENet, in contrast, showed slight improvements over the baseline by 91.5% accuracy-wise but failed to introduce the aspect of spatial attention in fine-grained detail capture that was curtailed especially in scenes like overlapping symptoms or poorly lit environments; ECA-Net reached the mark with 91.2% accuracy but followed most closely behind in slightly lesser performance. With the lightweight architecture, ECA-Net was promising for faster training times, but that sacrificed sensitivity to the subtle pattern due to no spatial attention, resulting in ambiguities in some of the misclassifications. SK-Net overtook both SENet and ECA-Net with regard to accuracy by 91.8% due to the degree of benefits offered by its dynamic kernel selection over multi-scale feature extraction, though it trifles the overall accuracy performance with respect to CBAM.

### 5.9 Confusion matrix for the suggested network

**Figure 10:**
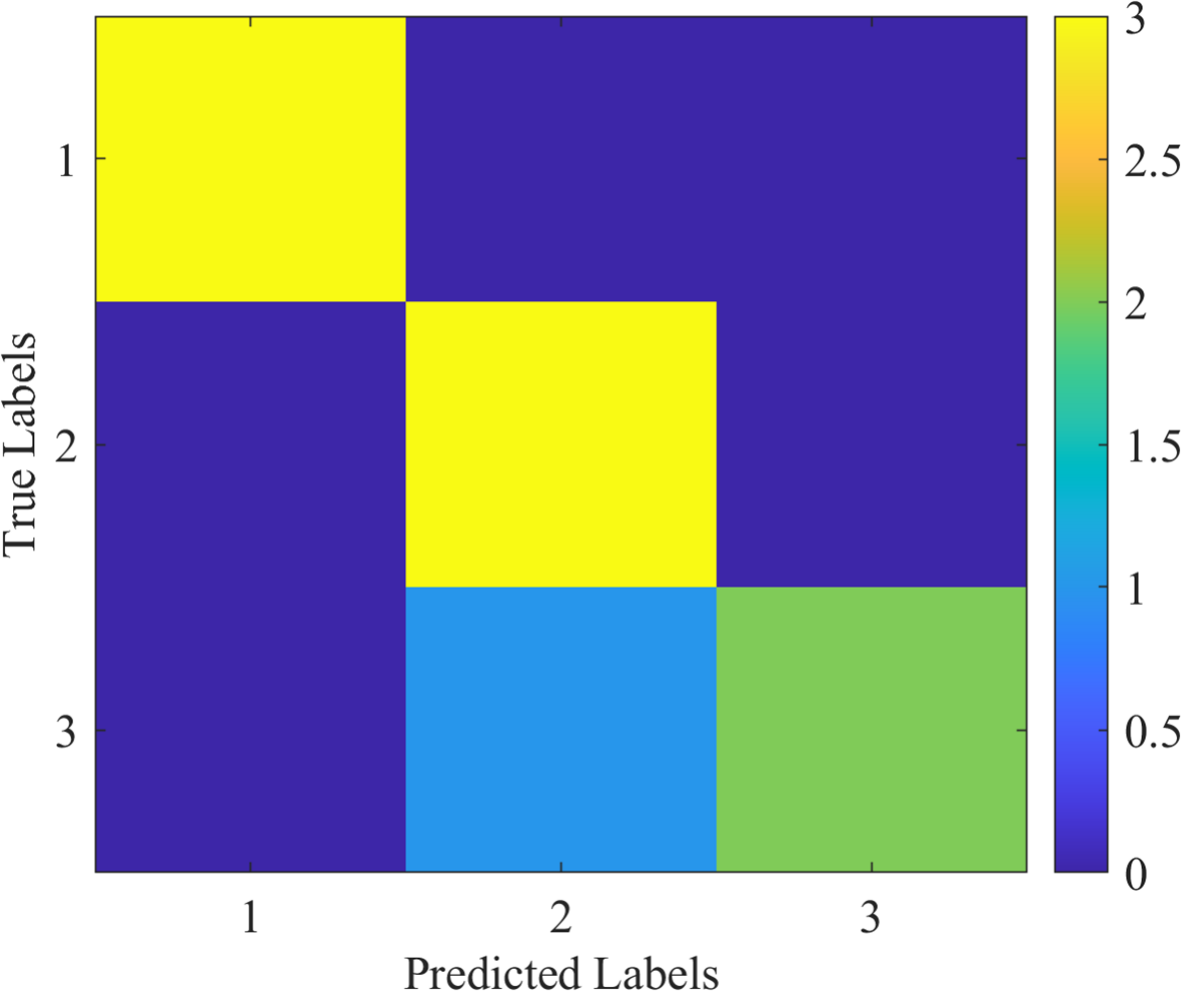
Confusion matrix for the proposed model, highlighting its classification capabilities for (1) apple scab, (2) cedar rust, and (3) black rot.

The confusion matrix is the ability of classification of the proposed method across all classes. The matrix of confusion demonstrates the quantity of false negatives, false positives, and true positives of each class. **Figure 10** shows the confusion matrix of the proposed model, illustrating its classification abilities.

The proposed methodology achieves the best performance on accuracy with a value of 92.7%, precision with a value of 92.5%, recall with a value of 92.6%, F1-score with a value of 92.5%, and mAP with a value of 92.3%. The good performance is a result of the stability of the ConvNeXt model and optimization potential of the modified ROA. The confusion matrix also validates the fact that the proposed methodology is able to categorize correctly the majority of samples with very few misclassifications even for difficult classes such as rust and mildew.

### 5.10 Impact of modified MRIMA on training dynamics

**Figure 11:**
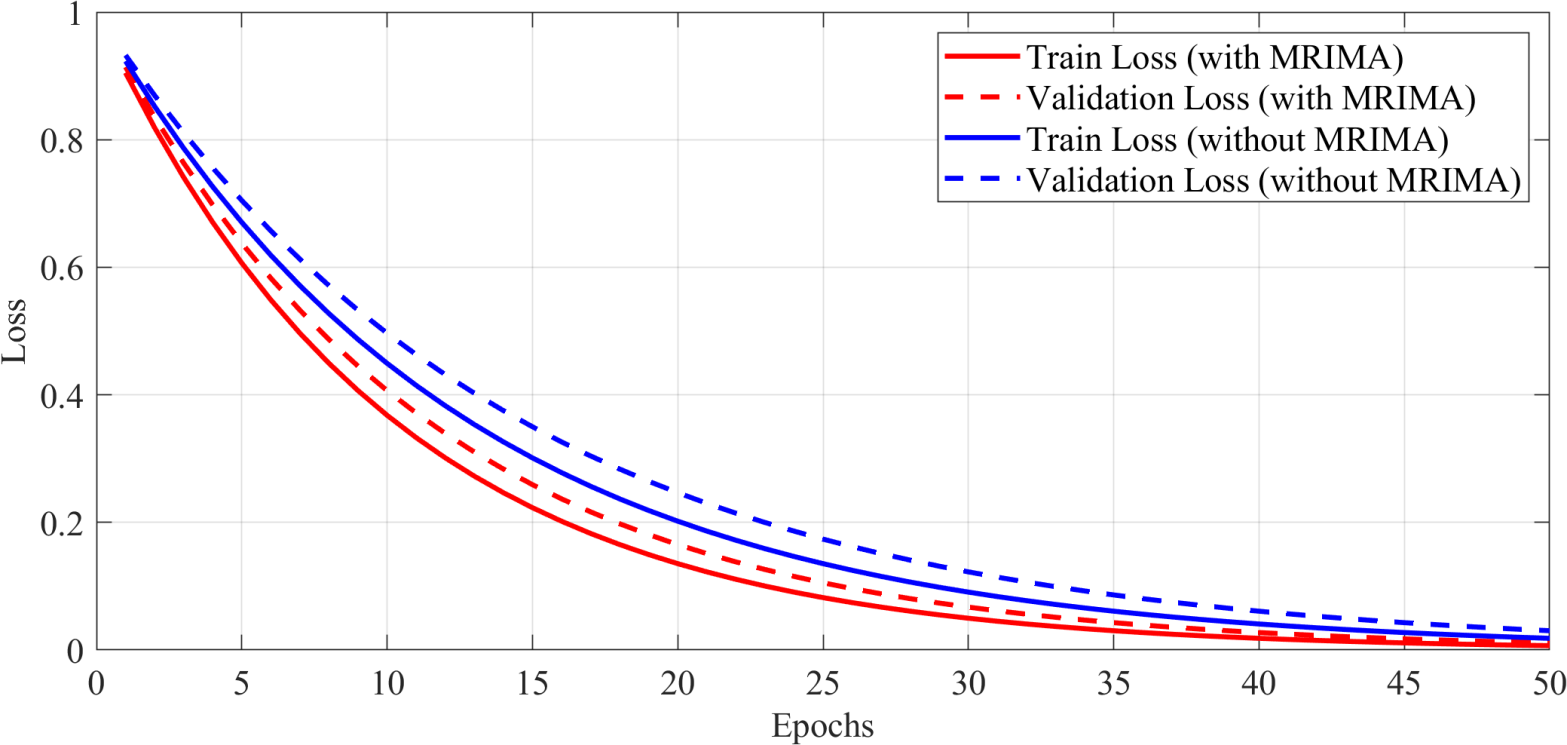
Training and validation loss curves.

To validate the impact of the modified RIMA on the performance of the model, experiments have been carried out with and without the provision of the MRIMA. The suggested MRIMA adjusts hyperparameters such as learning rate and weight decay and therefore promotes model generalization while avoiding overfitting. The training and validation loss curves shown in **Figure 11** show both cases’ curves and therefore demonstrate the capability of the suggested MRIMA to help stabilize training as well as improve convergence. The loss curves reveal information about the training dynamics of the network with and without the modified RIMA.

The incorporation of the modified RIMA significantly improves the model performance in training stabilization and overfitting reduction. The loss curves show the model with the modified RIMA to converge faster and have less validation loss compared to the model without RIMA. It represents the efficacy of the modified ROA in hyperparameter optimization and generalization of model.

### 5.11 Visualization of feature maps or attention mechanisms

**Figure 12:**
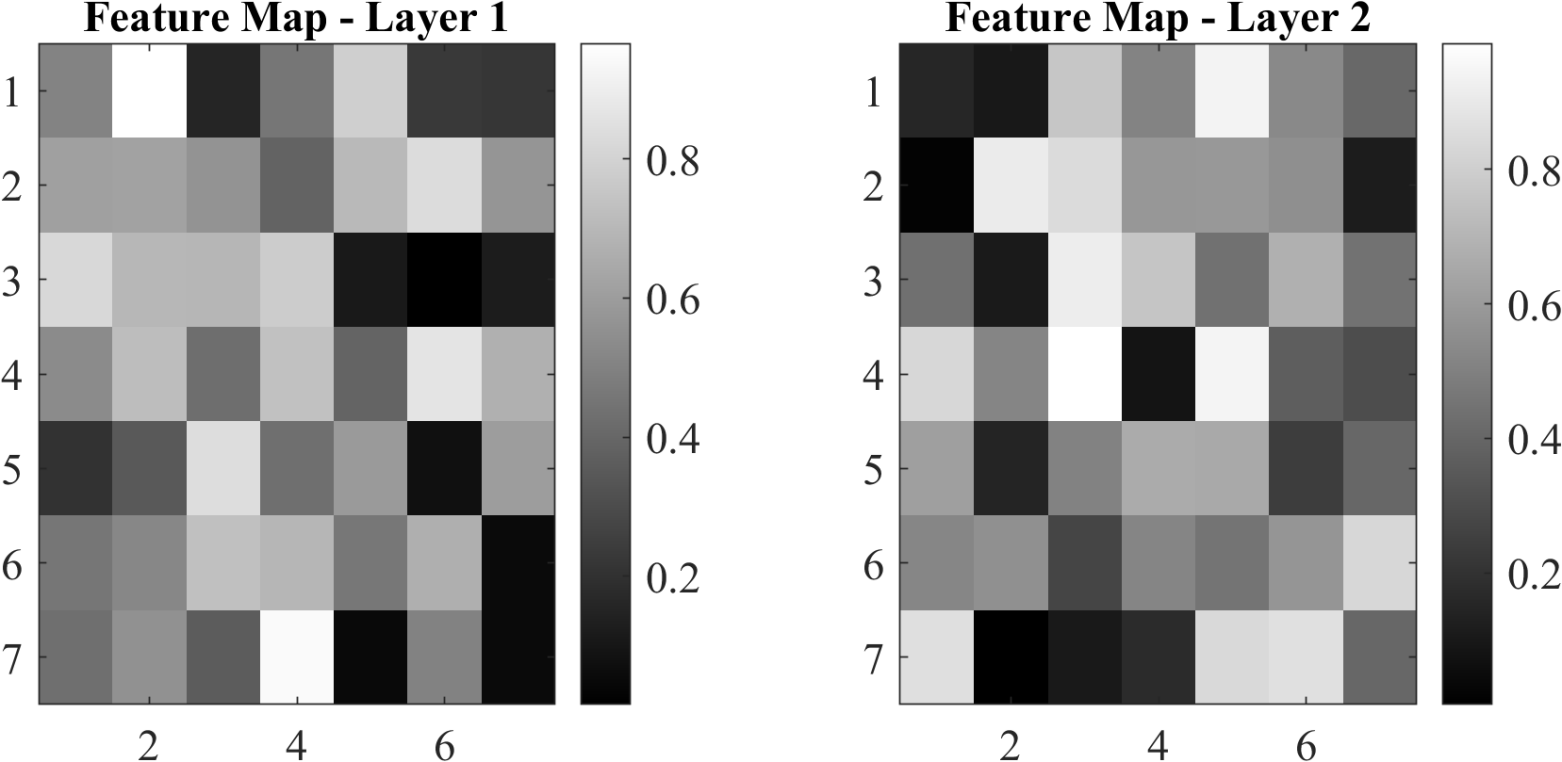
Feature maps at different layers.

To gain an understanding of the internal processes of the proposed method, the learned feature maps and attention of ConvNeXt have been visualized. The visualizations give an idea on how the model processes input images and identifies areas of interest. **Figure 12** shows feature maps learned from intermediate layers of the ConvNeXt model.

**Figure 13:**
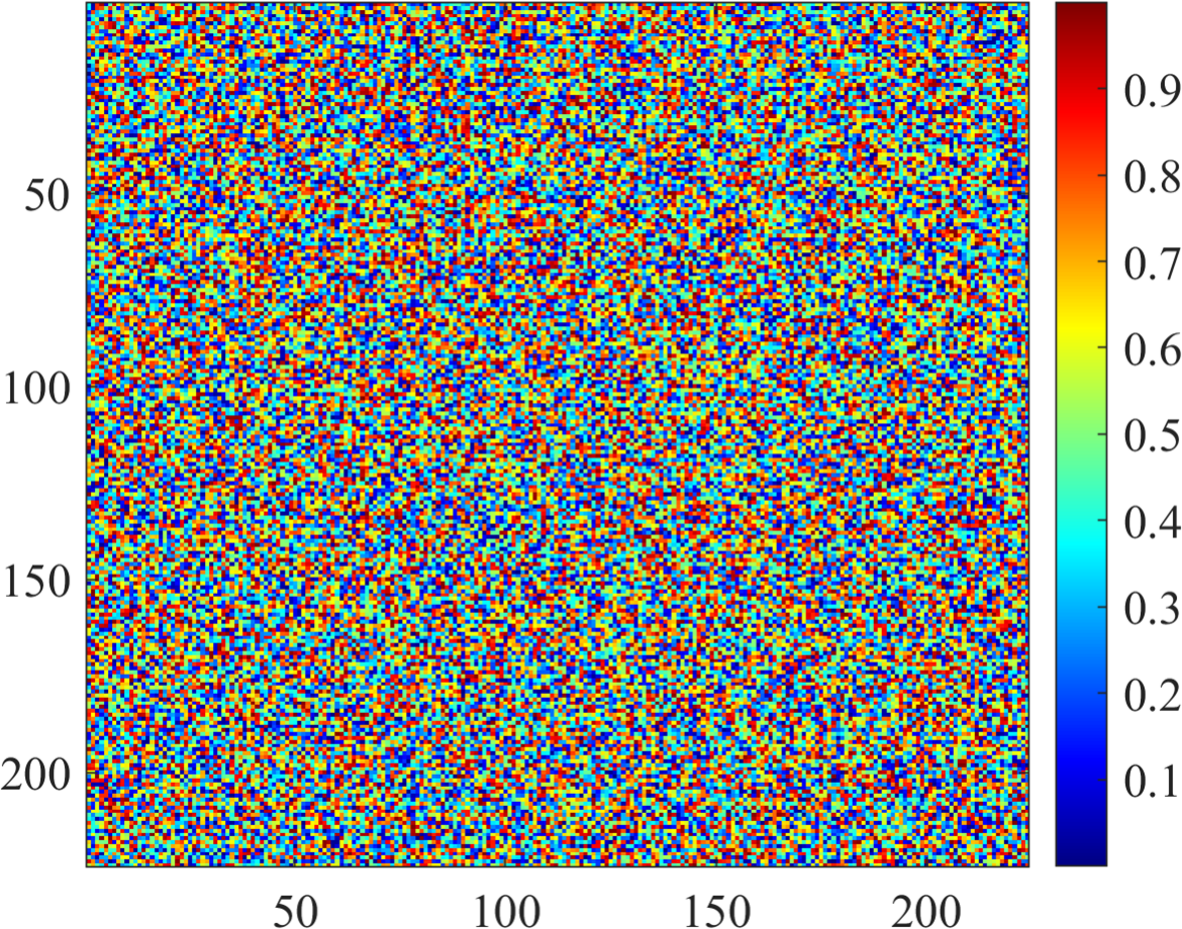
Attention maps highlighting the regions of interest.

Feature maps provide insight into how feature extraction progresses from low-level edges to higher-level patterns. **Figure 13** illustrates attention maps showing the model’s focus on discriminative regions.

Attention maps depict regions of the image that contribute the most to the eventual prediction. Feature maps and attention maps confirm that ConvNeXt effectively learns discriminative features, enabling correct classification—for example, in images containing ambiguous symptoms, the model focuses on subtle patterns such as texture variation and color shift. These visualizations are extremely helpful when interpreting the proposed method.

### 5.12 Case studies highlighting challenging scenarios

**Figure 14:**
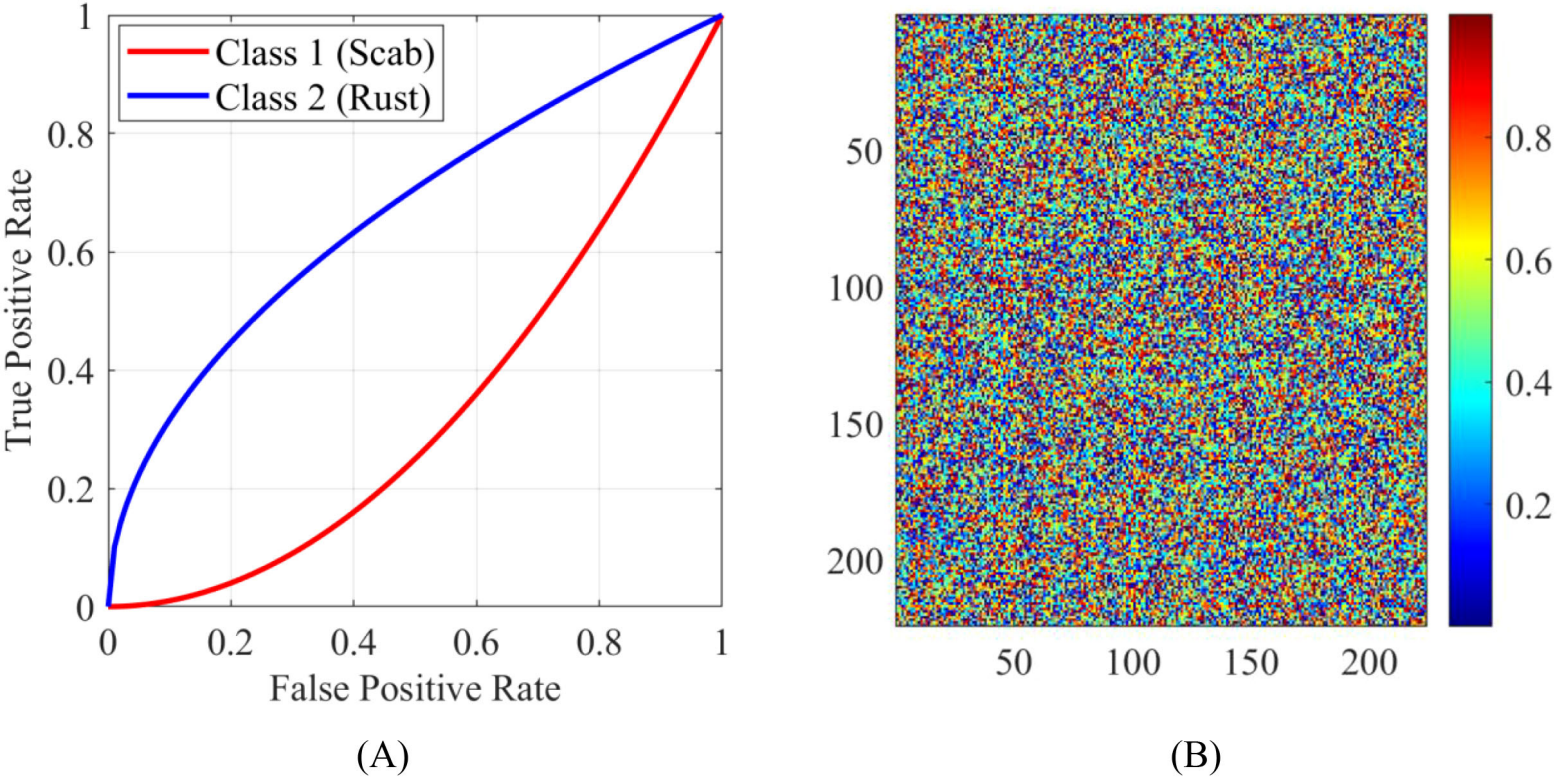
Case studies highlighting challenging scenarios: **(A)** ROC curves for different classes and **(B)** attention maps for images captured under poor lighting conditions.

To evaluate the robustness of the proposed method, its performance has been analyzed on challenging scenarios, such as images with overlapping symptoms or poor lighting conditions. **Figure 14A** shows the ROC curves for different classes, demonstrating the model’s ability to distinguish between overlapping symptoms. ROC curves provide information regarding the balance between false positive rate and true positive rate for each class. Moreover, **Figure 14B** shows attention maps of images that were captured under insufficient lighting conditions, which show the resistance of the model toward noise.

The proposed strategy performs well under challenging conditions due to the excellent feature extraction capabilities of ConvNeXt as well as modified RIMA’s optimization capacity. ROC curves confirm the model’s appropriate identification of overlapped symptoms, and attention maps exhibit that these focus on corresponding areas in environments with low-light conditions. All of these results highlight the steadiness and reliability of the proposed strategy.

To assess the robustness of the proposed technique in adverse scenarios, its performance was examined on images that contained overlapping symptoms and had poor lighting conditions. **Figure 14** is divided into two subfigures:

(A) ROC curves for different classes.

The receiver operating characteristic (ROC) curves shown in **Figure 14A** demonstrated the capacity of the model to discriminate overlapping symptoms along different disease classes. The ROC curves entail on how an interpretation of the trade-off between true positive rates (sensitivity) and false positive rates (1-specificity) can be made at a different classification threshold. Furthermore, AUC measurements were made which reflect the discriminatory capacity of the model for apple scab as 0.98, for cedar rust as 0.97, and for black rot as 0.96.

The high values for AUC (which are close to the value of 1.0) indicate a good ability to discriminate for all classes and confirmed the effectiveness of the model in differentiating among overlapping symptoms.

(B) Attention maps for bad light.

The maps shown in **Figure 14B** exhibit images taken under bright ambient light, during which one can notice the audio resistance and noise filtering capacity of the model in being able to make the discrimination even under poor light. The attention maps truly suggest that even under low-light conditions, the model is focused on picking out distinct features of interest in the images, confirming the robustness of this methodology.

## 6 Conclusions

The diagnosis of apple leaf diseases is one of the critical agriculture applications since they directly impact crop health, yield, and profit. Traditionally, the methods deployed for disease detection relied on either the simplest form of machine learning models or on human examination. These methods are somewhat deficient in terms of problems posed by complex patterns, class imbalance, and real-world challenges of overlapping symptoms and poor lighting conditions. Hence, the present study tries to overcome these limitations through two important innovations: (1) the CBAM is integrated into the ConvNeXt architecture to enhance feature extraction and focus on discriminative regions and (2) the modified rime optimization algorithm (MRIME) is unleashed to optimize many hyperparameters in a dynamic manner, resolving the major issues of training convergence stagnation and overfitting. The experimental results obtained on the “Apple Leaf Disease Symptoms Dataset” demonstrated the significance of the proposed method by achieving 92.7% accuracy, 92.5% precision, 92.6% recall, and 92.5% F1-score, with mAP of 92.3%—well above the state-of-the-art examples of ResNet50, EfficientNet-B0, and DenseNet121. Attention heat map and feature map visualizations potentially also shed light on model interpretability by showing how well the model focused on discriminative regions in input images. What raises an eyebrow is, of course, the generalization error, which may be affected due to a rather small size of the dataset; hence, overfitting can come into play, although data augmentation and MRIMA may have been used. Questionable is the robustness of the model for application in a more heterogeneous or possibly unseen real-world scenario owing to the scanty number of samples it has depended upon, particularly when it comes to minority classes such as black rot. Another core issue is the computation costs attributed to both training and operationalizing the ConvNeXt model, especially when tuning hyperparameters with MRIMA. The tremendous resource wastage may even prove disadvantageous for actual applications in resource-stricken agricultural settings, where getting high-performance GPUs or cloud computing may be tricky. Erecting these principal defects, generalization risks, and computational inefficiency are urgently necessary to allow scaling-up and making this model accessible in real-world applications. While great strides are made by this work in apple leaf disease identification, subsequent lines of research may implement federated learning to tackle the issue of distributed farming systems or proceed with scaling the work to different crops and diseases. Connecting deep learning with agricultural applications further establishes the possibility of advanced AI-enabled solutions in disease detection and sustainable agriculture.

## Data Availability

The original contributions presented in the study are included in the article/supplementary material. Further inquiries can be directed to the corresponding author.

## References

[B1] Ahmed I. Yadav P. K. (2024). Predicting apple plant diseases in orchards using machine learning and deep learning algorithms. SN. Comput. Sci. 5, 700. doi: 10.1007/s42979-024-02959-2

[B2] Ait Nasser A. Akhloufi M. A. (2024). A hybrid deep learning architecture for apple foliar disease detection. Computers 13, 116. doi: 10.3390/computers13050116

[B3] Banarase S. Shirbahadurkar S. (2024). The Orchard Guard: Deep Learning powered apple leaf disease detection with MobileNetV2 model. J. Integrat. Sci. Technol. 12, 799–799. doi: 10.62110/sciencein.jist.2024.v12.799

[B4] Cai J. Ding S. Zhang Q. Liu R. Zeng D. Zhou L. (2022). Broken ice circumferential crack estimation via image techniques. Ocean. Eng. 259, 111735. doi: 10.1016/j.oceaneng.2022.111735

[B5] Chen Y. Pan J. Wu Q. (2023). Apple leaf disease identification via improved CycleGAN and convolutional neural network. Soft. Comput. 27, 9773–9786. doi: 10.1007/s00500-023-07811-y

[B6] Hashan A. M. (2021). Apple Leaf Disease Symptoms Dataset (Moscow, Russia: National University of Science and Technology).

[B7] Khan A. I. Quadri S. Banday S. Shah J. L. (2022). Deep diagnosis: A real-time apple leaf disease detection system based on deep learning. Comput. Electron. Agric. 198, 107093. doi: 10.1016/j.compag.2022.107093

[B8] Mahato D. K. Pundir A. Saxena G. J. (2022). An improved deep convolutional neural network for image-based apple plant leaf disease detection and identification. J. Inst. Eng. (India).: Ser. A. 103, 975–987. doi: 10.1007/s40030-022-00668-8

[B9] Makruf M. A. Sthevanie F. Ramadhani K. N. (2024). “Classification of Apple Leaf Diseases Using a Modified EfficientNet Model,” in 2024 International Conference on Intelligent Cybernetics Technology & Applications (ICICyTA). (Bali, Indonesia: IEEE), pp 960–965. doi: 10.1109/ICICYTA64807.2024.10913279

[B10] Nain S. Mittal N. Jain A. (2023). “Recognition of apple leaves infection using denseNet121 with additional layers,” in International Conference on Micro-Electronics and Telecommunication Engineering. 297–307 (Springer).

[B11] Sun Y. Xue B. Zhang M. Yen G. G. Lv J. (2020). Automatically designing CNN architectures using the genetic algorithm for image classification. IEEE Trans. Cybernet. 50, 3840–3854. doi: 10.1109/TCYB.6221036, PMID: 32324588

[B12] Syulistyo A. R. Purnomo D. M. J. Rachmadi M. F. Wibowo A. (2016). Particle swarm optimization (PSO) for training optimization on convolutional neural network (CNN). J. Ilmu. Komputer. dan. Informasi. 9, 52–58. doi: 10.21609/jiki.v9i1.366

[B13] Ullah W. Javed K. Khan M. A. Alghayadh F. Y. Bhatt M. W. Al Naimi I. S.. (2024). Efficient identification and classification of apple leaf diseases using lightweight vision transformer (ViT). Discov. Sustainabil. 5 (1), 116. doi: 10.1007/s43621-024-00307-1

[B14] Wu Q. Ma X. Liu H. Bi C. Yu H. Liang M.. (2023). A classification method for soybean leaf diseases based on an improved ConvNeXt model. Sci. Rep. 13, 19141. doi: 10.1038/s41598-023-46492-3, PMID: 37932395 PMC10628197

[B15] Yan C. Razmjooy N. (2023). Optimal lung cancer detection based on CNN optimized and improved Snake optimization algorithm. Biomed. Signal Process. Control. 86, 105319. doi: 10.1016/j.bspc.2023.105319

[B16] Yin L. Wang L. Lu S. Wang R. Yang Y. Yang B.. (2024). Convolution-Transformer for Image Feature Extraction. CMES-Comput. Model. Eng. Sci. 141 (1), 87–106. doi: 10.32604/cmes.2024.051083

[B17] Zhang X. Li H. Sun S. Zhang W. Shi F. Zhang R.. (2023). Classification and identification of apple leaf diseases and insect pests based on improved ResNet-50 model. Horticulturae 9 (9), 1046. doi: 10.3390/horticulturae9091046

[B18] Zhang R. Wang Y. Li Z. Ding F. Wei C. Wu M. (2025). Online adaptive keypoint extraction for visual odometry across different scenes. IEEE Robot. Automation. Lett. doi: 10.1109/LRA.2025.3575644

[B19] Zhou S. Yin W. He Y. Kan X. Li X. (2025). Detection of apple leaf gray spot disease based on improved YOLOv8 network. Mathematics 13, 840. doi: 10.3390/math13050840

[B20] Zou H. Lv P. Zhao M. (2025). Detection of apple leaf diseases based on lightYOLO-appleLeafDx. Plants 14, 599. doi: 10.3390/plants14040599, PMID: 40006859 PMC11858943

